# Lysyl hydroxylase LH1 promotes confined migration and metastasis of cancer cells by stabilizing Septin2 to enhance actin network

**DOI:** 10.1186/s12943-023-01727-9

**Published:** 2023-01-31

**Authors:** Zihan Yang, Li Zhou, Tongxu Si, Siyuan Chen, Chengxi Liu, Kelvin Kaki Ng, Zesheng Wang, Zhiji Chen, Chan Qiu, Guopan Liu, Qingliang Wang, Xiaoyu Zhou, Liang Zhang, Zhongping Yao, Song He, Mengsu Yang, Zhihang Zhou

**Affiliations:** 1 Department of Biomedical Sciences, and Tung Biomedical Sciences Center, City University of Hong Kong, 83 Tat Chee Avenue, Kowloon, Hong Kong SAR, People’s Republic of China; 2 Department of Precision Diagnostic and Therapeutic Technology, City University of Hong Kong Futian Research Institute, Shenzhen, Guangdong China; 3 Department of Gastroenterology, the Second Affiliated Hospital of Chongqing Medical University, Chongqing, China; 4 State Key Laboratory of Chemical Biology and Drug Discovery, Research Institute for Future Food and Department of Applied Biology and Chemical Technology, The Hong Kong Polytechnic University, Hung Hom, Kowloon, Hong Kong SAR, China; 5 Department of Pathology, the Second Affiliated Hospital of Chongqing Medical University, Chongqing, China

**Keywords:** Confined migration, Lysyl Hydroxylase 1, Cancer metastasis, Septin2, Microfluidic chip

## Abstract

**Background:**

Excessive extracellular matrix deposition and increased stiffness are typical features of solid tumors such as hepatocellular carcinoma (HCC) and pancreatic ductal adenocarcinoma (PDAC). These conditions create confined spaces for tumor cell migration and metastasis. The regulatory mechanism of confined migration remains unclear.

**Methods:**

LC–MS was applied to determine the differentially expressed proteins between HCC tissues and corresponding adjacent tissue. Collective migration and single cell migration microfluidic devices with 6 μm-high confined channels were designed and fabricated to mimic the in vivo confined space. 3D invasion assay was created by Matrigel and Collagen I mixture treat to adherent cells. 3D spheroid formation under various stiffness environment was developed by different substitution percentage GelMA. Immunoprecipitation was performed to pull down the LH1-binding proteins, which were identified by LC–MS. Immunofluorescent staining, FRET, RT-PCR, Western blotting, FRAP, CCK-8, transwell cell migration, wound healing, orthotopic liver injection mouse model and in vivo imaging were used to evaluate the target expression and cellular phenotype.

**Results:**

Lysyl hydroxylase 1 (LH1) promoted the confined migration of cancer cells at both collective and single cell levels. In addition, LH1 enhanced cell invasion in a 3D biomimetic model and spheroid formation in stiffer environments. High LH1 expression correlated with poor prognosis of both HCC and PDAC patients, while it also promoted in vivo metastasis. Mechanistically, LH1 bound and stabilized Septin2 (SEPT2) to enhance actin polymerization, depending on the hydroxylase domain. Finally, the subpopulation with high expression of both LH1 and SEPT2 had the poorest prognosis.

**Conclusions:**

LH1 promotes the confined migration and metastasis of cancer cells by stabilizing SEPT2 and thus facilitating actin polymerization.

**Supplementary Information:**

The online version contains supplementary material available at 10.1186/s12943-023-01727-9.

## Background

Desmoplasia, the formation of dense collagenous stroma, is a strong indicator of tumor aggressiveness and poor clinical outcome in cancer patients [1]. The fibrogenic stroma and stiff extracellular matrix (ECM) not only result from tumor growth but also play essential roles in cellular transformation and tumor progression [2]. Metastasis is the leading cause of cancer-related deaths, and it is a complicated cascade consisting of local invasion, intravasation, survival of harsh conditions in circulation, extravasation, and colonize at a distant site. It is known that metastatic liver tumor tissues are stiffer than primary colorectal cancer tissues, and reducing stiffness of metastatic liver cancer by angiotensin-converting enzyme inhibitors improves the survival time of metastatic colorectal cancer patients [3]. Moreover, it has been reported that the reduction of tissue stiffness by tumor suppressor gene Ras association domain family member 1 (RASSF1A) inhibited metastatic dissemination [4]. Cancer cells migrate in vivo by degrading the surrounding ECM to create their own tracks or by moving through pre-existing channel-like tracks. The ECM contains confined pores (ranging from 1 μm to 20 μm in diameter) or channel-like tracks varying from less than 3 μm to 30 μm wide and 100 μm to 600 μm in length [5]. Confined migration happens not only during local invasion but also during intravasation, extravasation, and metastatic foci establishment. For example, it has been shown that breast cancer cells that could pass through a membrane with pores 3 μm in diameter had a marked advantage to form lung metastasis [6]. Thus, targeting the confined migration might help interrupt the metastatic cascade. Currently, the confined migration environments that cancer cells encounter in vivo can be recaptured in vitro by biomimetic three dimensional (3D) ECM gels, microfluidic structures, or transwell chambers [7, 8].

Both hepatocellular carcinoma (HCC) and pancreatic ductal adenocarcinoma (PDAC) are characterized by obvious desmoplasia [9, 10]. Liver cancer is ranked the sixth most common cancer worldwide and the second leading cause of cancer death [11]. HCC is the predominant histologic type and occurs for 85% of liver cancer. There is growing evidence indicating that collagen deposition and crosslinking can worsen tumor progression by promoting cancer cell migration and invasion [12]. PDAC is a highly aggressive disease with a 5-year survival rate of < 10% [13]. The remarkable ECM stiffness and desmoplasia surrounding PDAC cells constitute an anatomically supporting tissue and facilitate tumor growth, metastasis, and survival [14]. Therefore, we applied HCC and PDAC, the two different cancer types to this study due to their similar characteristics of stiff environment.

The lysyl hydroxylase (LH) family includes three members, LH1, LH2 and LH3, and as the name indicates, it is mainly responsible for lysyl hydroxylation. Based on the immunofluoresence staining results in the Protein Atlas database, LH family members are predicted to be located in endoreticullum, extracellular space, nucleus, and cytoskeleton, but there lacks exact evidence to reveal their subcellular localization. It has been reported that LH1 and LH2 specifically function as a lysyl hydroxylase, promoting cross-linking in ECM molecules, and contributing to ECM structural stability and maturation [15]. Recent reports show that the LH family plays an important role in tumor progression. LH2 can promote invasion and metastasis of breast cancer, non-small cell lung cancer, ovarian cancer, and osteosarcoma [16, 17]. The expression level of LH2 seems to positively correlate with the grade of HCC and poor prognosis. LH3 can promote the occurrence of liver cancer, and is found highly expressed in ovarian cancer tissues. Recent studies show that LH1 was highly expressed in glioblastoma tissues and promoted cell proliferation and invasion [18]. In addition, LH1 was also found to be highly expressed in osteosarcoma tissue and positively correlated with poor prognosis [19, 20]. Bioinformatic studies also showed that high LH1 expression was positively related with the poor prognosis of HCC [21] and PDAC patients [22]. However, the role of LH1 in confined migration and metastasis of HCC and PDAC is poorly known.

Septins, the fourth component of the cytoskeleton other than microfilaments, microtubules, and intermediate fibers, are a family of multimeric GTPases which assemble into non-polar polymers [23, 24]. The Septin family contains 13 members in total, which can self-assemble into hexamers (Septin2-6–7-7–6-2) or octamers (Septin2-3–6-7–7-6–3-2) to further assemble into filamentous structures [25, 26]. Septins physically reinforce the actomyosin network by crosslinking actin filaments, readying cells for the higher mechanical demands of migration that include elevated contractility, mechanotransduction, and mechanical stress [27]. The molecular regulation of Septin network remains poorly known.

In the present study, we found using proteomic analysis that LH1 was overexpressed in HCC tissues. High LH1 expression correlated with poor prognosis of both HCC and PDAC patients. We designed and applied microfluidic devices to study the effect of LH1 in confined migration of HCC and PDAC cells. The results showed that LH1 significantly promoted the confined migration of cancer cells at both collective and single cell levels, with no effect on the proliferation and unconfined migration. In addition, we revealed that LH1 could enhance cell invasion in the 3D biomimetic model, spheroid formation in stiffer environment and in vivo metastasis. At the molecular level, we found that LH1 could bind and stabilize one cytoskeleton component, Septin 2 (SEPT2), which further enhanced the polymerization of the actin network. The hydroxylase domain of LH1 protein was essential for the interaction between LH1 and SEPT2. Finally, we demonstrated the positive correlation between LH1 and SEPT2 expression level in both HCC and PDAC tissues, and showed that the subpopulation with high expression of LH1 and SEPT2 has the poorest prognosis. The current work revealed novel molecular mechanism of LH1 on confined migration and metastasis of HCC and PDAC, which may provide a new strategy for cancer diagnosis and treatment.

## Methods

### Cell lines and cell culture

Human liver cancer cell lines HUH7, Hep3B, PLC/PRF/5, SK-Hep1, SNU-449, HepG2, pancreatic cancer cell line SW1900 and PANC1, lung cancer cell line H69 and A549, breast cancer cell line MDA231, and cervical cancer cell line Hela were obtained from the American Type Culture Collection (ATCC, USA) and cultured with Dulbecco’s Modified Eagle Medium (DMEM, Gibco, Thermo Fisher, USA). All cultured media were supplemented with 10% fetal bovine serum (FBS) and 1% penicillin–Streptomycin (10,000 Units/mL of penicillin and 10000 µg/mL of streptomycin, Life Technologies, USA). All cell lines were in the incubator with 5% CO_2_ at 37 ℃ (CO_2_ water-jacketed incubator, Nuaire®, USA). All cells were identified by short-tandem-repeat profiling and without mycoplasma contamination.

### Clinical samples

HCC specimens from 153 patients and PDAC specimens from 63 patients were collected from 2011 to 2015 at the Second Affiliated Hospital of Chongqing Medical University after informed consent was obtained from all patients. The patients did not receive chemotherapy or radiotherapy before surgery. The diagnoses of HCC or PDAC were made independently by at least two histopathologists. This study was carried out according to the principles of the Helsinki Declaration and approved by the Ethical Committee of the Second Affiliated Hospital of Chongqing Medical University ((2019)133).

### Proteomics analysis

Eight paired HCC tissues and adjacent tissues were subjected to tandem mass tags (TMT) proteomic analysis (Luming Biotechnology, China). The samples were extracted, lysed, and marked with TMT, and then prepared for Liquid chromatography-mass spectrometer (LC–MS) detection. The differentially expressed proteins were further used for enrichment analysis. For the immunoprecipitation samples, the obtained spectral raw data were analyzed by the software Proteome Discoverer 2.2 using the Sequest search engine. The reference Homo sapiens proteomes, last modified on March 7, 2021, were downloaded from UniProt.

### Fluorescence resonance energy transfer (FRET)

Cells were transfected with lentivirus expressing the fusion gene hLH1-3xGGGGS-GFP and hSEPT2-3xGGGGS-mCherry. Cells with stable expression were screened with puromycin (LH1) and G418 (SEPT2). FRET was done by a Nikon confocal microscopy (A1HD25, Japan) using 60 × plan apochromatic oil immersion objective. The FRET channel was acquired by excitation at 488 nm and a 561 nm emission filter or vice versa. To exam the protein with GFP, it set as donor channel with exication at 488 nm and acceptor channel with a 561 nm emission filter. Cells transfected with either hLH1-3xGGGGS-GFP or hSEPT2-3xGGGGS-mCherry were used as negative control.

### Microfluidic chip fabrication and usage

The confined microfluidic device was designed by AutoCAD and fabricated the device by traditional photolithography method. Master mold was developed on silicon wafers with 4 μm and 24 μm by using SU-8 3005 and 3050 (Kayakuam, Japan) with 2500 rpm and 3000 rpm. We molded poly(dimethylsiloxane) (PDMS) (10:1 silicone elastomer with curing agent, Sylgard 184, Dow Corning®, USA), followed with 15 min degas and 2 h 65℃ curation, then peeled out the pattern and punched 2 mm holes at channel inlets and outlets. Two PDMS layers were treated by air plasma (Plasma cleaner/sterilizer, PPC-3XG, Harrick®, USA) and bonded together under the microscope with the alignment to create 4 μm confined migration space. The device was exposed to ultraviolet light for 45 min for sterilization, then treated the device channel with 2% Fibronectin (Thermo Fisher, USA) under 37℃ incubations for strength cell adhesion. Then we added cells into channel inlet and performed real-time tracking of cells for 48 h and captured images every 30 min (LS720, Etaluma, USA).

### Vertical invasion of tumor cells into ECM

The vertical invasion model was slightly modified according to previous work [28]. 1 × 10^4^ cells were added into 96-well plates in triplicate and allowed 24 h for complete cell adhesion. We then removed the medium and added 50µL ECM 50% Matrigel (Corning, USA) and 50% Collagen I mixture (Thermo Fisher, USA) to cells and cultured for 48 h to allow cells to vertically invade. We then scanned for layers by using a high content instrument (ImageXpress Micro, Molecular Device, USA) for a total of 100 μm with 10 μm interval. We then counted cell numbers for each layer by a high content analysis system to compare the invasion ability between different sample groups.

### 3D cell culture under various stiffness GelMA model

We prepared 5% (w/v) and 10% (w/v) GelMA solution by using 60% and 98% substitutional rate solid GelMA with 0.5% lithium acylphosphonate photo-initiator (LAP) (EFL, China). We then exposed 100 µL of solution to the ultraviolet light source (1.35 W/cm^2^) for 40 s and measured the hydrogel stiffness by rheometer (DMA Q800, TA instrument, USA) to get 292 Pa and 4695 Pa hydrogels. 4000 cells were added to 50 µL GelMA solution and seeded into 96-well plates. We then shook the plate until the GelMA solution covered the whole bottom of the well and exposed the plate to UV light source for curation. We added 100 µL fresh medium to each well and incubated for 5 min for GelMA gelation liquids removal. We replaced the medium with 150 µL fresh complete medium for 14 days of culture. Then, we captured images of generated spheroid by high content instrument (ImageXpress Micro, Molecular Device, USA), followed by high content analysis [29].

### Immunoprecipitation (IP) and Co-IP

Pierce™ Classic Magnetic IP/Co-IP Kit was applied to the immunoprecipitation and co-immunoprecipitation. Briefly, the specific antibody was first added to the cell lysate to form an immune complex that was then bound to the magnetic beads. The complex was washed to remove non-bound material and a low pH elution buffer dissociated the bound immune complex from the Protein A/G. The immune complex was then applied to subsequent mass spectrometry or Western blot.

### Liver injection in vivo model

1 × 10^6^ tumor cells (SK-Hep1-Vector and SK-Hep1-LH1; or SK-V-Ctrl, SK-V-sh2, SK-LH1-Ctrl, SK-LH1-sh2) with luciferase-expression were suspended in 50 μl 50wt% Matrigel (Corning®, Cat#356,231, USA) and injected into SCID mice liver directly. The in vivo tumors were monitored weekly using the IVIS. The mice were sacrificed 8 weeks after injection and the liver and lung tissues were collected for pathological test. All animals used in this work are approved by City University of Hong Kong ethical committee (A-0588).

### Fluorescence recovery after photobleaching (FRAP)

This experiment was done according to a previous study [30]. The cells that had stable β-actin-mCherry fusion protein expression were transferred to 35 mm glass-bottom cell culture dishes (Nest, China). Imaging was performed using the Nikon A1HD25 confocal microscope. In brief, three frames were imaged using low laser power and a rectangular region was subsequently bleached with 8 iterations at full laser power. Imaging was performed with 16 s time-lapse intervals for 180 s. Fluorescence recovery then followed with identical settings to prebleach/activation frames. Fluorescence intensity was measured from small areas of the bleached region. Recovery rate was calculated according to the formula: recovery rate = (Vt-Vpost)/(Vpre-Vpost), where Vt is the intensity at the indicated time point, Vpre is the intensity before bleaching, and Vpost is the intensity right after bleaching.

### Statistical analysis

All values are presented as mean ± standard deviation (SD). All data were obtained from at least three repetitions of each experiment. Prism 8.0 (GraphPad, USA) and SPSS 18.0 software were used to analyze the data. All data are shown as mean ± SD. Student’s t-test was used to analyze the differences between two groups with normal distribution. The Mann–Whitney analysis was applied to compare ranks in nonparametric data. One-way ANOVA was used to compare three or more groups. Kaplan–Meier plot was applied to analyze the patient survival time. KMplotter and UALCAN were used for online analysis of the TCGA data. Probability values less than 0.05 was considered statistically significant.

## Results

### High LH1 expression correlates with poor prognosis of HCC and PDAC patients

**Fig. 1:**
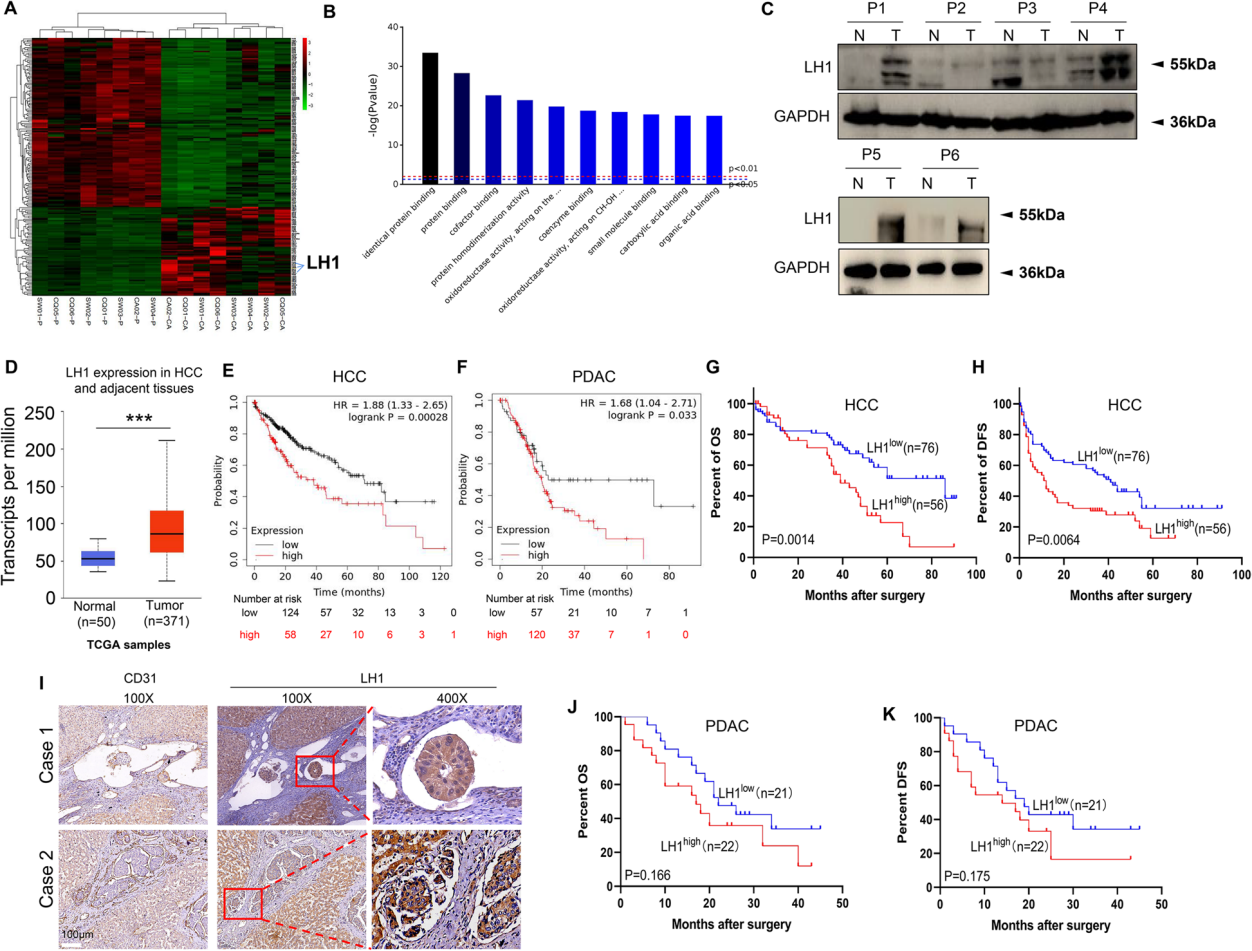
High LH1 expression correlates with poor prognosis of HCC and PDAC patients** A** Heat map of the differentially expressed proteins between 8 paired HCC tissues and adjacent tissues via proteomic analysis. **B** GO molecular function analysis of the differentially expressed proteins. **C** Protein expression level of LH1 in HCC and adjacent tissues by the Western blot. **D** LH1 mRNA expression level in HCC tissues from TCGA database. **E**&**F** Kaplan–Meier survival analysis showing that patients with high LH1 expression level had shorter overall survival (OS) time in HCC (E), and PDAC (F) using KMplotter database. **G**&**H** Patients with high LH1 protein expression level had shorter OS time (G) and disease-free survival (DFS) time (H) in HCC patients (n = 132) by Kaplan–Meier survival analysis. **I** IHC staining showing the LH1 expression in HCC tumor embolus. **J**&**K** Patients with high LH1 protein expression level had shorter OS time (J) and DFS time (K) in PDAC patients (*n* = 43) via Kaplan–Meier survival analysis

Based on proteomic analysis of eight HCC tissues and corresponding adjacent tissues, we found that LH1 was among the most significantly overexpressed proteins in HCC tissues (Fig. 1A, Table 1). Other elevated proteins in HCC tissues, such as POSTN [31, 32], S100P [33], ACSL4 [34], MCM6 [35], MCM4 [36], SMAP [37], PLIN2 [38], G6PD [39, 40], MCM2 [41], have been revealed to regulate hepatocarcinogenesis (Supplementary Table 1). The gene ontology (GO) analysis showed that the differentially expressed proteins were enriched in molecular functions such as identical protein binding, cofactor binding, and protein homodimerization (Fig. 1B). We further confirmed that LH1 was highly expressed in HCC tissues using the Western blot (Fig. 1C). Based on The Cancer Genome Atlas (TCGA) data, LH1 seems to be highly expressed in HCC tumors (*n* = 371) (Fig. 1D).

**Table 1:** The association between LH1 or SEPT2 expression and clinicopathological parameters

Features	No. of patients (%)	LH1 expression status		*P*	SEPT2 expression status		*P*
		**Low (*n* = 92) No.patient (%)**	**High (*n* = 61) No.patient (%)**		**Low (*n* = 80) No.patient (%)**	**High (*n* = 73) No.patient (%)**	
Gender							
Male	131(85.1%)	78(59.5%)	53(40.5%)	0.717	67(51.1%)	64(48.9%)	0.490
Female	23(14.1%)	14(63.6%)	8(40.5%)		13(59.1%)	9(40.9%)	
Age							
≤ 52	83(%)	49(59.0%)	34(41.0%)	0.763	15	39(47.0%)	0.845
> 52	71(%)	43(61.4%)	27(38.6%)		36(51.4%)	34(48.6%)	
Number							
1	124(%)	75(60.5%)	49(39.5%)	0.313	62(50.0%)	62(50.0%)	0.079
2	12(%)	9(75.0%)	3(25.0%)		10(83.3%)	2(16.7%)	
3 or more	17(%)	8(47.1%)	9(52.9%)		8(47.1%)	9(52.9%)	
Tumor size(cm)							
≤ 3	54(%)	35(64.8%)	19(35.2%)	0.382	28(51.9%)	26(48.1%)	0.936
> 3	100(%)	57(57.6%)	42(42.4%)		52(52.5%)	47(47.5%)	
Tumor size(cm)							
≤ 5	92(%)	57(62.0%)	35(38.0%)	0.571	45(48.9%)	47(51.1%)	0.305
> 5	62(%)	35(57.4%)	26(42.6%)		35(57.4%)	26(42.6%)	
Macrovessel invasion							
No	138(%)	87(63.0%)	51(37.0%)	0.026	75(54.3%)	63(45.7%)	0.122
Yes	15(%)	5(33.3%)	10(66.7%)		5(33.3%)	10(66.7%)	
Distant metastasis							
0	140(%)	87(62.1%)	53(37.9%)	0.095	73(52.1%)	67(47.9%)	0.906
1	13(%)	5(38.5%)	8(61.5%)		7(53.8%)	6(46.2%)	
BCLC stage							
0	25(%)	20(80.0%)	5(20.0%)	0.010	15(60.0%)	10(40.0%)	0.038
1	91(%)	56(61.5%)	35(38.5%)		45(49.5%)	46(50.5%)	
2	13(%)	8(61.5%)	5(38.5%)		11(84.6%)	2(15.4%)	
3	24(%)	8(33.3%)	16(66.7%)		9(37.5%)	15(62.5%)	
Differentiation degree							
Poor	30(%)	19(63.3%)	11(36.7%)	0.525	20(66.7%)	10(33.3%)	0.025
Moderate	103(%)	59(57.3%)	44(42.7%)		46(44.7%)	57(55.5%)	
Well	20(%)	14(70.0%)	6(30.0%)		14(70.0%)	6(30.0%)	
Microinvasion							
Negative	136(%)	84(61.8%)	52(38.2%)	0.243	75(55.1%)	61(44.9%)	0.045
Positive	17(%)	8(47.1%)	9(52.9%)		5(29.4%)	12(70.6%)	
SEPT2 expression status							
low	80(%)	63(78.8%)	17(21.3)	0.000	-	-	-
high	74(%)	29(39.2%)	45(60.8%)		-	-	-

We then analyzed the prognostic value of LH1 in pan-cancer types by using KM-plotter. The results showed that high LH1 expression was associated with unfavorable prognosis of HCC (Fig. 1E), PDAC (Fig. 1F), stomach adenocarcinoma, and lung adenocarcinoma (Supplementary Fig. 1A and B) patients. Among patients with sorafenib treatment, the patients with higher LH1 expression had only half of the survival rate as the low LH1 expression group (Supplementary Fig. 1C). Higher grade of cancer showed a higher LH1 expression level (Supplementary Fig. 1D). We further confirmed the unfavorable prognostic value of LH1 in 153 HCC patients by immunohistochemical (IHC) staining. We found that high LH1 expression significantly correlated with macrovasular invasion and the Barcelona Clinic Liver Cancer (BCLC) stage (Table 1). The representative IHC stain images are shown in Supplementary Fig. 1E. We confirmed that high LH1 protein expression level was associated with short overall (39 months vs. 86 months) (Fig. 1G) and disease-free survival time (11.5 months vs. 41 months) (Fig. 1H). Interestingly, we found that LH1 was also strongly expressed in the tumor embolus (Fig. 1I). Coincidentally, PDAC patients with high LH1 expression had shorter overall median survival time (17 months vs. 22 months) (Fig. 1J) and disease-free survival time (median survival time: 14 months vs 19 months) (Fig. 1K). The tumor size was larger in LH1^high^ subgroup than LH1^low^ group among PDAC patients (Supplementary Fig. 1F). In summary, we revealed that LH1 expression level was associated with macrovascular invasion, and poor prognosis in HCC and PDAC patients.

### LH1 promotes confined migration from multidimensional levels

**Fig. 2:**
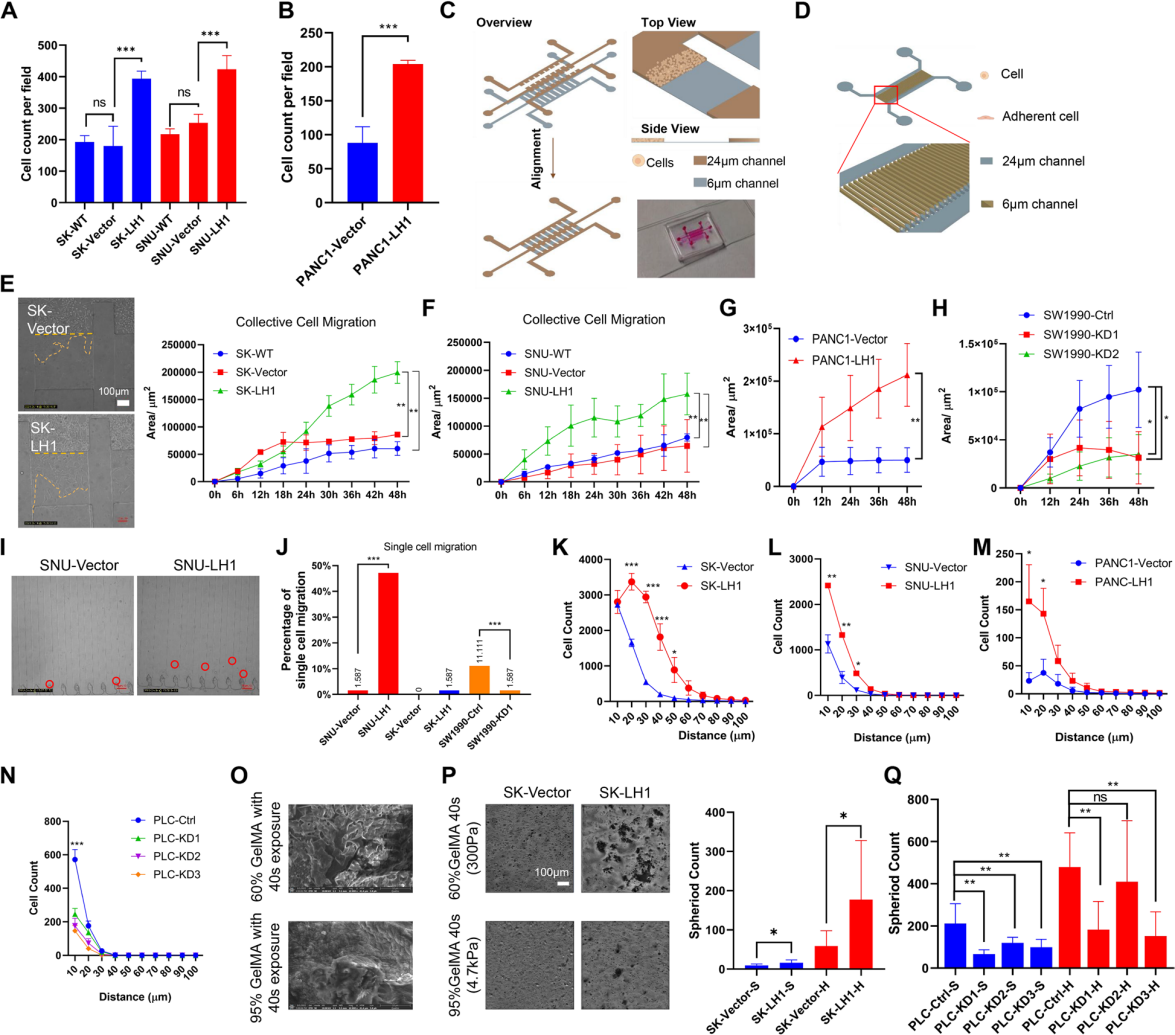
LH1 overexpression promotes confined migration on multidimensional levels** A** Transwell migration of wild type (WT), control (Vector), and LH1-overexpressing SK-Hep1 and SNU449 cells. **B** Transwell migration of control (Vector) and LH1-overexpressing PANC1 cells. **C** Schematic view of the collective confined migration chip. **D** Schematic image of single cell confined migration chip. **E–G** 2D confined migration of wild type (WT), control (Vector), and LH1-overexpressing SK-Hep1 (E), SNU449 cells (F), and PANC1 cells (G). **H** 2D confined migration of control (Ctrl) and LH1-knockdown SW1990 cells. **I** Images showing the single cell confined migration in the chip of SNU449 cells. **J** Single cell confined migration percentage of LH1-overexpressing cells or LH1-knockdown cells. **K-N** Statistical results of 3D invasion of LH1-overexpressing SK-Hep1 (K), SNU449 (L), PANC1 (M), or LH1-knockdown PLC/PRF/5 cells (N). **O** The scanning electron microscope images of 60% or 95% substitutional GelMA with 40 s exposure. **P** 3D sphere formation of SK-Hep1 cells with or without LH1 overexpression. **Q** Knock down of LH1 suppressed the 3D invasion of PLC5 cells in stiff gels. *, *P* < 0.05;**, *P* < 0.01;***, *P* < 0.001

Based on the RT-PCR and Western blot results in HCC, PDAC, lung cancer, and breast cancer, we found that LH1 was highly expressed in PLC/PRF/5 (HCC) and SW1990 (PDAC) cells, and its expression was low in SK-Hep1 (HCC), SNU449 (HCC), and PANC1 (PDAC) cells (Supplementary Fig. 2A and B). Both HCC cells and PDAC cells were transfected with lentiviruses expression the fusion gene hLH1-3xGGGGS-GFP to visualize the distribution of LH1. Consistent to the prediction that LH1 can be located in endoreticullum, extracellular space, nucleus, and cytoskeleton by the Protein Atlas database, we observed that LH1 was not specifically distributed to endoreticullum (Supplementary Fig. 2C). We then overexpressed or knocked down LH1 via lentivirus transfection in these cells with low or high LH1 expression respectively to test its function (Supplementary Fig. 3A-D). We did not find significant change in colony formation (Supplementary Fig. 3E and F), cell proliferation (Supplementary Fig. 3G and H), or unconfined 2D migration (wound healing) (Supplementary Fig. 3I and J). However, we found that LH1 overexpression could significantly promote the HCC (Fig. 2A) or PANC1 cells to pass through the 8 μm pores in the transwell migration assay (Fig. 2A and B), while knockdown of LH1 inhibited the migration of PLC/PRF/5 cells (Supplementary Fig. 4A). This result implied that LH1 might promote the confined migration of cancer cells.

We then fabricated microfluidic chips with 6 μm-high migration channels for both the collective confined migration (Fig. 2C) and single cell confined migration (Fig. 2D) to study the effect of LH1 on confined migration [42]. We found that LH1 overexpression significantly enhanced the collective confined migration speed of HCC cells SK-Hep1 and SNU449 (Fig. 2E and F; Supplementary Fig. 4B, Supplementary Video 1) and PANC1 PDAC cells (Fig. 2G). PLC/PRF/5 cells failed to enter the confined migration channel due to self-cluster formation after incubation, but knockdown of LH1 could reduce the confined migration of SW1990 (Fig. 2H) and SK-Hep1 cells (Supplementary Fig. 4C). We further found that LH1 overexpression could promote the confined migration of SNU449 cells at the single cell level, and knocking down LH1 reduced the single cell confined migration of SW1990 cells. SK-Hep1 cells could hardly migrate into the single cell confined migration channel (Fig. 2I&J, Supplementary Fig. 4D and E).

The confined migration ability was also examined on 3D models. We detected the effect of LH1 on 3D invasion by using a mixture of Matrigel and type I collagen to create an invasion environment above adherent cells [28]. The results showed that LH1 overexpression could increase the 3D invasion capacity of HCC cells (Fig. 2K and L, Supplementary Fig. 4F) and PDAC cells (Fig. 2M). Consistently, knockdown of LH1 dampened the 3D invasion of PLC/PRF/5 (Fig. 2N), and SK-Hep1 cells (Supplementary Fig. 4G). Finally, we applied a stiff 3D model to mimic the in vivo environment [29]. Using the 95% substitutional rate GelMA, the stiffness could reach 4.7 kPa (Supplementary Fig. 4H), which is close to the tumor tissue stiffness [43]. Under scanning electron microscope examination, the stiffer gel structure was observed to be more intense (Fig. 2O). We found that LH1-overexpressed cells generated more spheroids under higher GelMA stiffness environment (Fig. 2P and Supplementary Fig. 4I), while knocking down LH1 could reduce the sphere formation of HCC cells (Fig. 2Q and Supplementary Fig. 4J). To further validate the function of LH1, we applied the LH1 inhibitor dipyridyl [44] to test its effect, which is the only reported LH1 inhibitor with limited specificity. The results showed that dipyridyl could significantly decrease the confined migration and 3D invasion of the LH1-overexpressed cells, but this effect was slight on control cells (Supplementary Fig. 4K-N). Overall, we demonstrated that LH1 could promote the confined migration of both HCC and PDAC cells at multidimensional levels.

### LH1 binds to SEPT2 and enhances actin polymerization

**Fig. 3:**
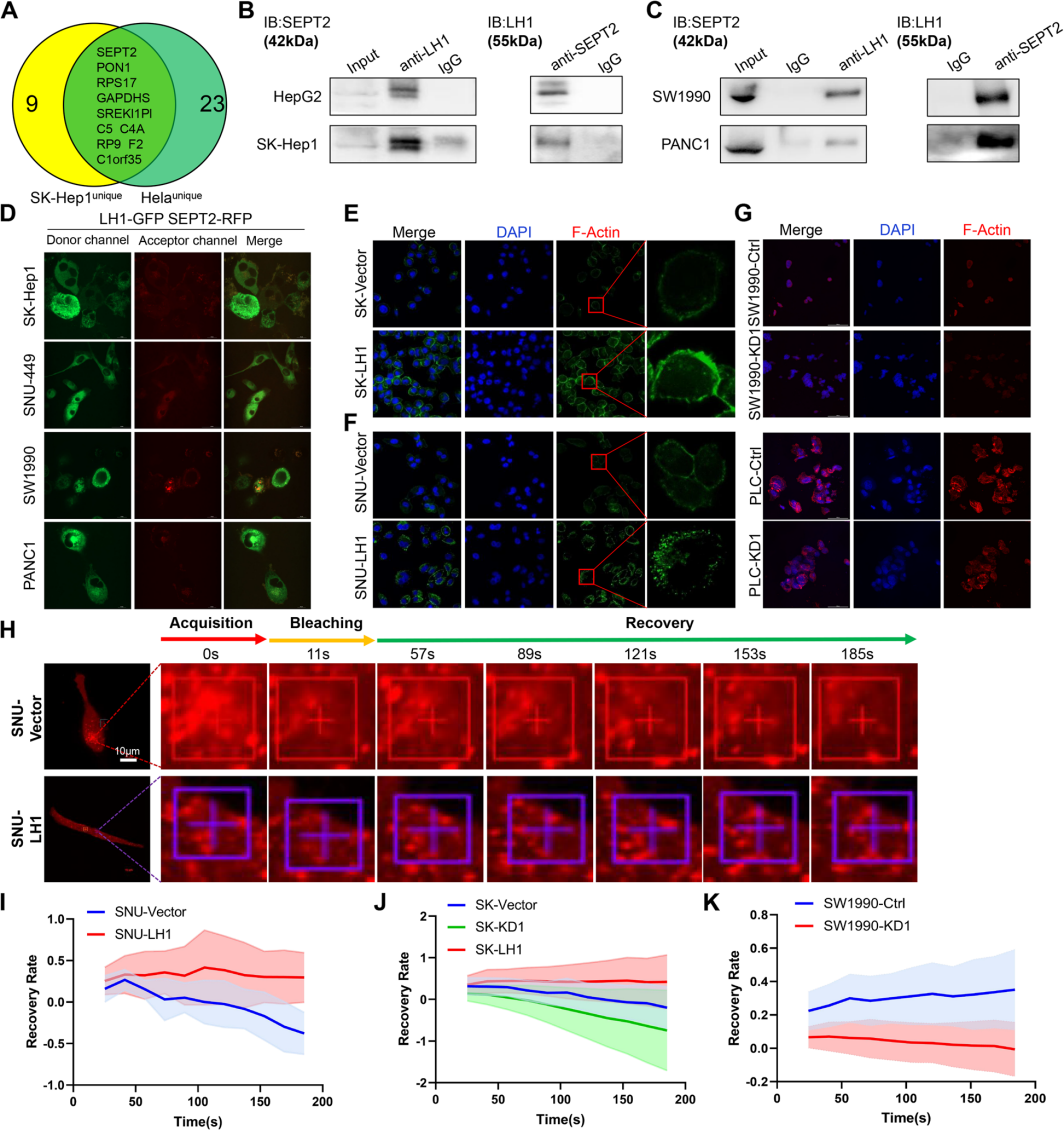
LH1 binds with SEPT2 and enhances actin polymerization **A** Venn diagram of the unique LH1-binding proteins in SK-Hep1 and Hela cells. **B**&**C** Co-immunoprecipitation and reverse immunoprecipitation showing the binding between LH1 and SEPT2 in different HCC cell lines (B) and PDAC cells (C). D FRET showing the interactions between LH1 and SEPT2 among HCC and PDAC cell lines. **E**&**F** Immunofluorescence showing F-actin in LH1-overexpressing SK-Hep1 (E) and SNU449 (F) cells. **G** Immunofluorescence showing F-actin in LH1-knockdown SW1990 (*upper panel*) and PLC/PRF/5 (*lower panel*) cells. **H** Representative FRAP images at indicated time points of LH1-overexpressing or control SNU449 cells. **I-K** Statistical results of FRAP assays in SNU449 (I), SK-Hep1(J), and SW1990 (K) cells

To reveal the mechanism by which LH1 regulates HCC cell confined migration, we obtained protein complexes binding to LH1 by co-immunoprecipitation in SK-Hep1 cells and Hela cells. Subsequent mass spectrometry analysis showed that LH1 could specifically bind to filament-related protein SEPT2 (Fig. 3A), which belong to the “fourth cytoskeleton” septin family [24] and has been reported to promote the migration and invasion of HEK293 cells [45]. Interestingly, our results did not reveal the binding between LH1 and collagen, which has been reported to be modified by LH family members. Further co-immunoprecipitation experiments confirmed that LH1 and SEPT2 protein could bind together in both HCC cells (Fig. 3B) and PDAC cells (Fig. 3C). Immunofluorescence staining also showed that LH1 co-localized with SEPT2 in both HCC cells and PDAC cells (Supplementary Fig. 5). We applied the FRET assay to further validate the binding between LH1 and SEPT2. The FRET phenomenon can be observed when the cells were transfected lentiviruses expression fusion proteins hLH1-3xGGGGS-GFP and hSEPT2-3xGGGGS-mCherry (Fig. 3D). In contrast, the cells only expressing hLH1-3xGGGGS-GFP or hSEPT2-3xGGGGS-mCherry did not show the FRET phenomenon (Supplementary Fig. 6A and B). Recent studies have shown that septin family molecules could also regulate microfilaments composed of filamentous actin (F-actin) [25]. We found that LH1 can enhance the formation of F-actin network in HCC cells (Fig. 3E and F), while knockdown of LH1 led to the reduction of F-actin network (Fig. 3G). Fluorescence recovery after photobleaching (FRAP) was applied to explore the polymerization of actin using mCherry-actin-expressing lentivirus. The recovery rate was positively associated with the actin polymerization [30]. We further showed that LH1 overexpression promoted the actin polymerization (Fig. 3H-J), while LH1 knock down reduced the actin polymerization (Fig. 3K). Consistently, we found that the LH1-overexpressing cells formed more F-actin network than control cells in the confined migration channels (Supplementary Fig. 6C-E). Thus, these results demonstrated that LH1 could bind with SEPT2 and enhance the structure of microfilaments.

### LH1 stabilizes SEPT2 using its hydroxylase function

**Fig. 4:**
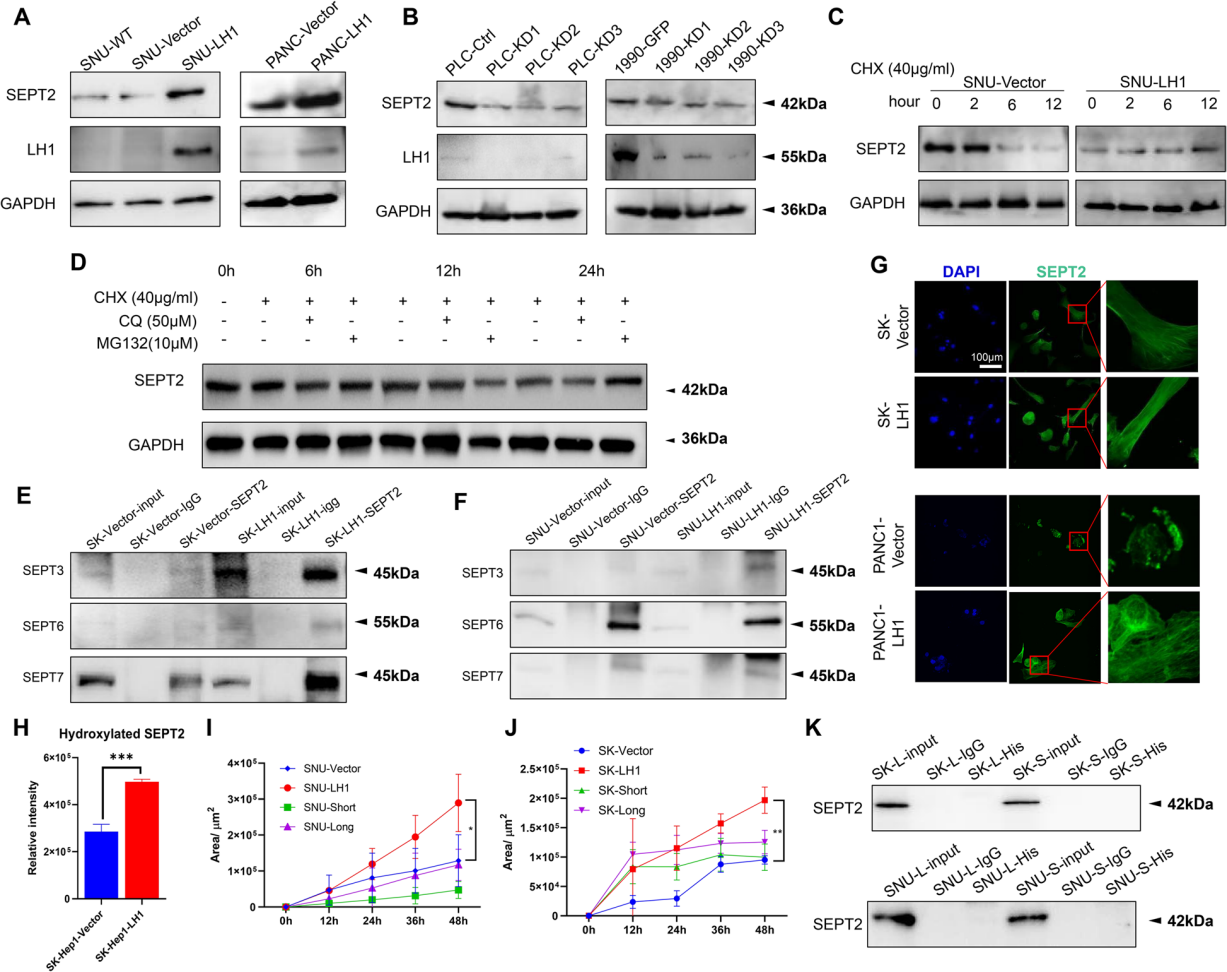
LH1 stabilizes SEPT2 using its hydroxylase function** A** Western blot showing the upregulation of SEPT2 by LH1 overexpression in SK-Hep1 and SNU449 cells. **B** Western blot showing the upregulation of SEPT2 by LH1 overexpression in PANC1 cells. **C** Western blot showing the downregulation of SEPT2 by LH1 knockdown in PLC/PRF/5 and SK-Hep1 cells. **D** Western blot showing the chloroquine treatment could reduce the degradation of SEPT2 protein. **E**&**F** Co-immunoprecipitation showing the binding of SEPT2 to SEPT3/6/7 in control and LH1-overexpressing SK-Hep1 (E) and SNU449 (F) cells. **G** Immunofluorescence showing the septin network in LH1-overexpressing SK-Hep1 and PANC1 cells. **H** Statistic result of hydroxylation level of SEPT2 protein in LH1 vector and overexpression group. **I**&**J** Confined migration of SNU449 (I) or SK-Hep1 (J) cells transfected with LH1 truncations. **K** Co-immunoprecipitation with 6 × His antibody showed that the truncated LH1 proteins could not bind with SEPT2. *, *P* < 0.05;**, *P* < 0.01;***, *P* < 0.001

Septins are regarded as the “forth type of cytoskeleton molecules”. They can self-assemble into hexamers (septin2-6–7-7–6-2) or octamers (septin2-3–6-7–7-6–3-2), and further assemble into filamentous structures [25]. However, the regulatory mechanism of septin network remains unclear. Herein, we found that the mRNA level of SEPT2 was not affected by LH1 expression (Supplementary Fig. 7A). On the other hand, LH1 overexpression could increase the protein abundance of SEPT2 in HCC and PDAC cells (Fig. 4A, Supplementary Fig. 7B). On the other hand, knockdown of LH1 led to the downregulation of SEPT2 (Fig. 4B, Supplementary Fig. 7B). These results implied posttranslational modification of SEPT2 expression. We then measured the degradation of SEPT2 proteins using cycloheximide (CHX) to block protein synthesis. We found that LH1 could reduce the degradation of SEPT2 proteins in SNU449 (Fig. 4C) and SK-Hep1 (Supplementary Fig. 7C) cells. To determine whether the proteosome or the lysosome pathway mediates the degradation of SEPT2, we treated the cells with corresponding inhibiors, MG132 or chloroquine. We found that chloroquine treatment could stabilize the SEPT2 protein (Fig. 4D), implying that LH1 stbilized SEPT2 by inhibiting the lysosome-mediated degradation. Moreover, LH1 overexpression increased the binding between SEPT2 and other septin family members (septin3/6/7) (Fig. 4E and F). Immunofluorescence staining showed that LH1 could enhance septin network (Fig. 4G) while knocking down LH1 suppressed the septin network in both HCC and PDAC cells (Supplementary Fig. 7D and E). We further found that LH1 could increase the hydroxylation of SEPT2 protein by mass-spectrum analysis (Fig. 4H, Supplementary Fig. 8A).

Furthermore, we generated a long truncation (L) in which the hydroxylase domain was depleted and a short truncation (S) which lost both hydroxylase and glycotranferase domain (Supplementary Fig. 8B). These truncations failed to induce confined migration of HCC cells (Fig. 4I&J), and consistently failed to immunoprecipitate SEPT2 proteins (Fig. 4K). Taken together, these results revealed that LH1 hydroxylates and stabilizes SEPT2 protein to enhance the septin network, depending on the hydroxylase domain.

### Knockdown of SEPT2 reverses the effect of LH1

**Fig. 5:**
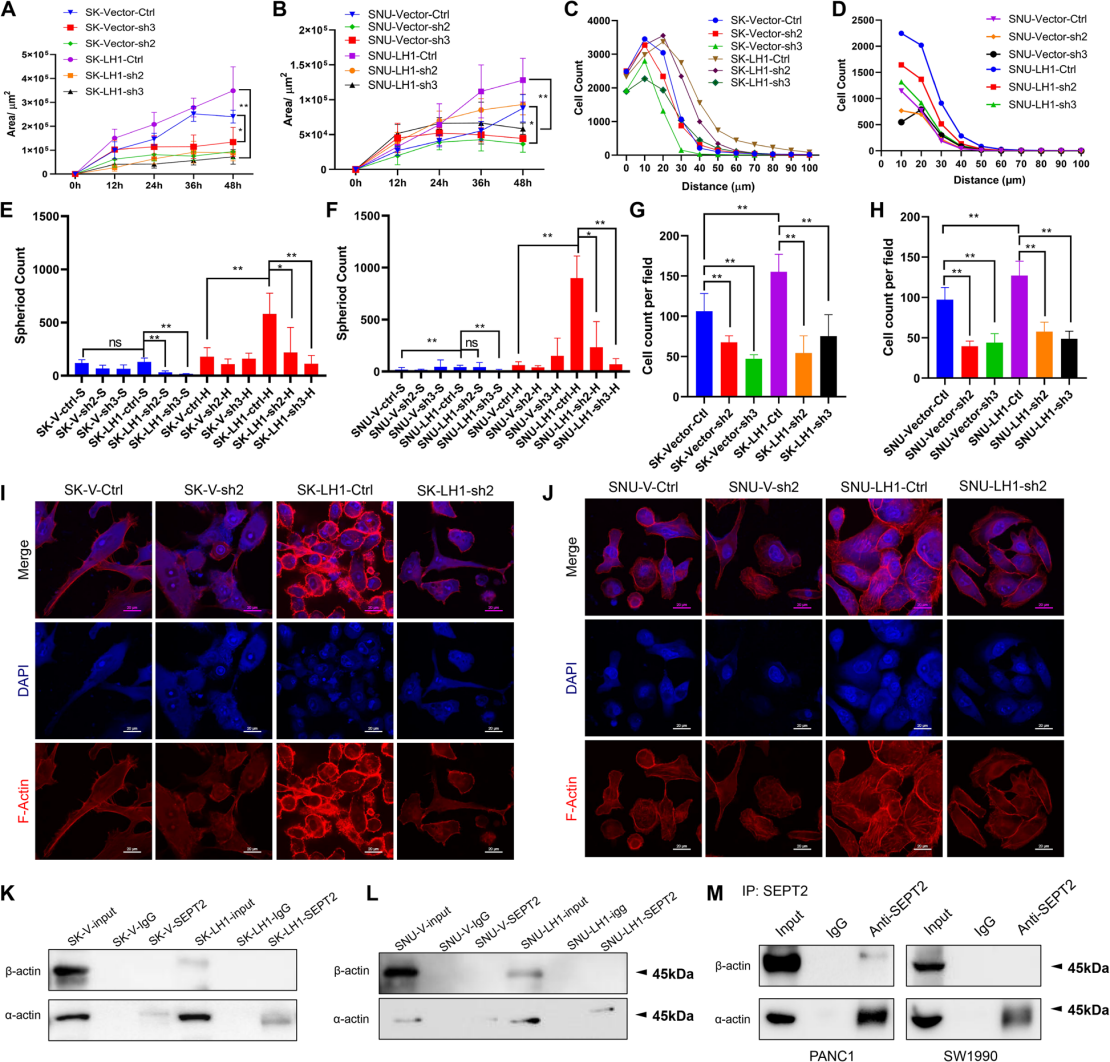
Knockdown of SEPT2 reverses the effect of LH1** A**&**B** Knockdown of SEPT2 (assigned as sh2 or sh3) inhibited the confined migration of LH1-overexpressing SK-Hep1 (A) and SNU449 (B) cells. **C** Knockdown of SEPT2 reversed the 3D invasion of LH1-overexpressing SK-Hep1 cells. Significant difference was found between SK-Vector-Ctrl and SK-Vector-sh3 cells, and between SK-LH1-Ctrl and SK-LH1-sh3 cells. **D** Knockdown of SEPT2 reversed the 3D invasion of LH1-overexpressing SNU449 cells. Significant difference was found between SNU-LH1-Ctrl and SNU-LH1-sh2 or sh3 cells. **E**&**F** Knockdown of SEPT2 reduced the sphere formation of LH1-overexpressing SK-Hep1 (E) and SNU449 (F) cells, which was more obvious under hard hydrogels. **G**&**H** Transwell migration showing that knockdown of SEPT2 reduced the transmembrane migration of LH1-overexpressing SK-Hep1 (G) and SNU449 (H) cells. **I**&**J** Knockdown of SEPT2 led to the reduction in actin filaments in LH1-overexpression SK-Hep1 (I) and SNU449 cells (J). **K**&**L** Co-immunoprecipitation assays showing the binding between SEPT2 and α-actin but not β-actin in SK-Hep1 (K) and SNU449 cells (L). Overexpression of LH1 enhanced the binding. **M** Co-immunoprecipitation assays showing the binding between SEPT2 and α-actin but not β-actin in PDAC cells. *, *P* < 0.05;**, *P* < 0.01;***, *P* < 0.001

We then tested whether SEPT2 mediated the function of LH1 in confined migration by knocking down SEPT2 in both control cells and LH1-overexpressing cells (Supplementary Fig. 9A and B). We found that knocking down SEPT2 could significantly inhibit the collective confined migration of both control and LH1-overexpressing cells (Fig. 5A and B). Furthermore, knocking down LH1 could diminish the gap between control and LH1-overexpressing cells. Coincidently, suppressing SEPT2 expression reduced the 3D invasion of cancer cells, especially the LH1-overexpressing cells (Fig. 5C and D). In the stiff sphere formation model, we also found that SEPT2-knockdown cells could form less sphere, which was more obvious in hard gels (Fig. 5E and F). The transwell migration assay also showed that knocking down SEPT2 could remarkably suppress cell migration (Fig. 5G and H, Supplementary Fig. 9C and D). Additionally, we found that SEPT2-knockdown SK-Hep1 and SNU449 cells had less F-actin network and knockdown of SEPT2 could diminish the effect of LH1 on F-actin intensity (Fig. 5I and J). Co-immunoprecipitation assay revealed that SEPT2 could bind with α-actin but not β-actin; this was enhanced by LH1 overexpression (Fig. 5K and L). We could also detect the binding between SEPT2 and α-actin in PDAC cells (Fig. 5M). Similar to LH1, SEPT2 also had no significant effect on unconfined 2D migration (Supplementary Fig. 9E and F). These data revealed that SEPT2 mediated the function of LH1 in confined migration by regulating the polymerization of actin proteins.

### LH1 promotes in vivo metastasis via SEPT2

**Fig. 6:**
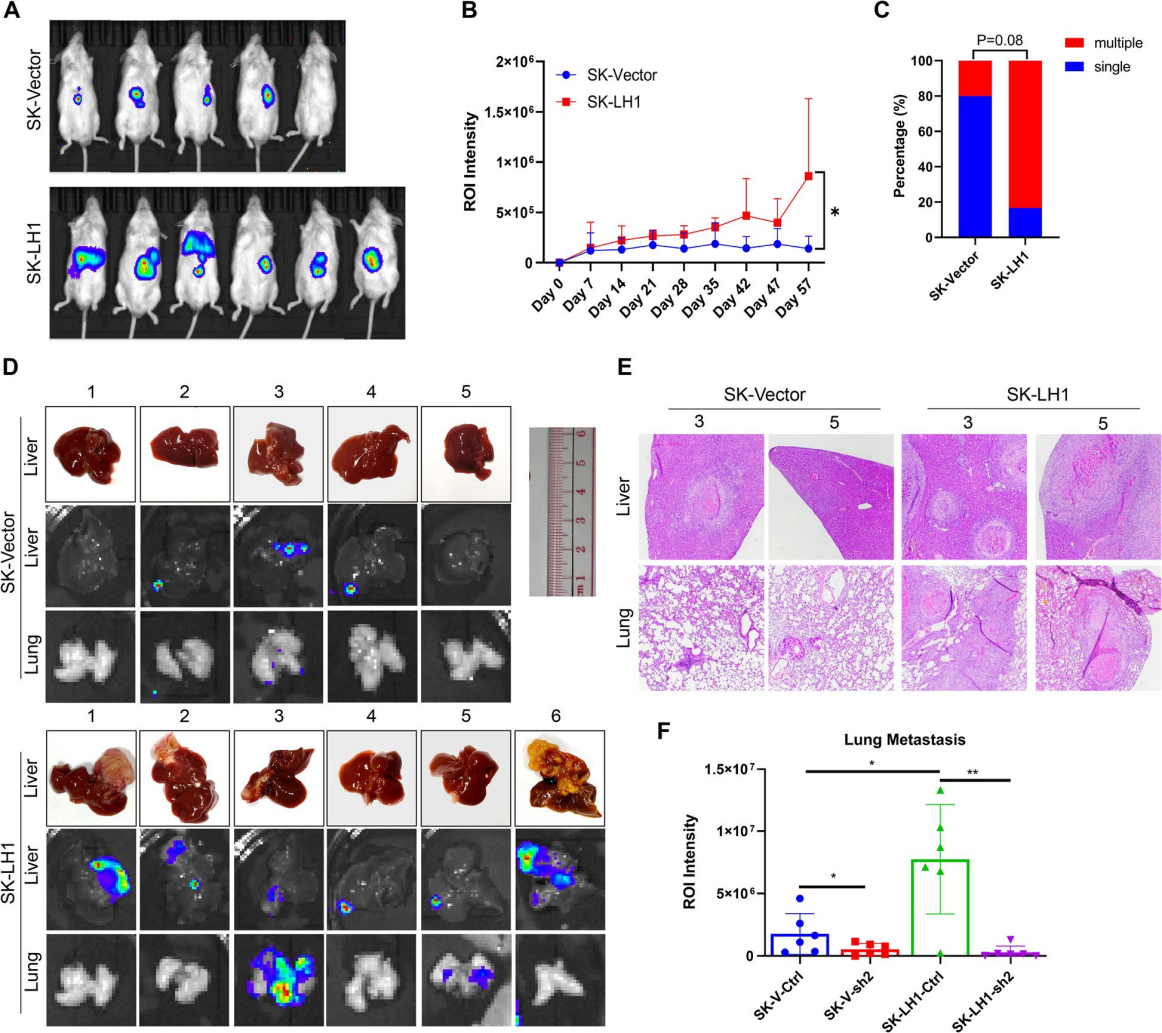
LH1 promotes in vivo metastasis via SEPT2 **A** In vivo imaging of the tumor-bearing mice at the end of the experiment. **B** Bioluminescence intensity of the control or LH1-overexpressing group at the indicated time point. **C** Percentage of mice with multiple-centered tumor (more than 1 tumor center or distant metastasis). **D** Gross image of liver under bright field and bioluminescence imaging of each group. **E** HE staining showing the tumor in liver or lung of each group. **F** Bioluminescence intensity of the lung metastasis among LH1 vector, overexpression and SEPT2 knockdown groups. *, *P* < 0.05;**, *P* < 0.01;***, *P* < 0.001

Confined migration occurs during cancer metastasis. To test the in vivo function of LH1 on tumor metastasis, we adopted the liver orthotopic injection model. LH1-overexpressing and vector SK-Hep1 cells were injected into the liver of non-obese severe diabetes mellitus with immune deficiency (NOD-SCID) mice. We found that LH1-overexpressing cells could form larger tumor mass (Fig. 6A and B). Most of the tumors originating from LH1-overexpressing cells developed more than one nodule (Fig. 6C), implying intrahepatic or extrahepatic metastasis. The livers and lungs were collected at the end of the experiment. We observed that the LH1-overexpressing group had significantly larger tumors in the liver, and two of them developed lung metastatic foci (Fig. 6D). Hematoxylin & eosin (HE) staining confirmed that more liver tumors and lung metastasis were formed in the LH1-overexpressing group (Fig. 6E). Finally, IHC staining showed that both LH1 and SEPT2 were highly expressed in primary tumor and metastatic foci (Fig. 6F, Supplementary 10A), indicating that high LH1 expression subgroup could easily form tumors. Consistently, we knocked down SEPT2 in both LH1 vector and overexpression group and found out that knock-down of SEPT2 dramatically reduced the lung metastasis in LH1-overexpression cells, with slight effect in control cells (Supplementary 10B). On the whole, we demonstrated that LH1 could promote the in vivo metastasis of HCC cells.

### Co-expression of LH1 and SEPT2 correlates with the poorest prognosis of HCC and PDAC patients

**Fig. 7:**
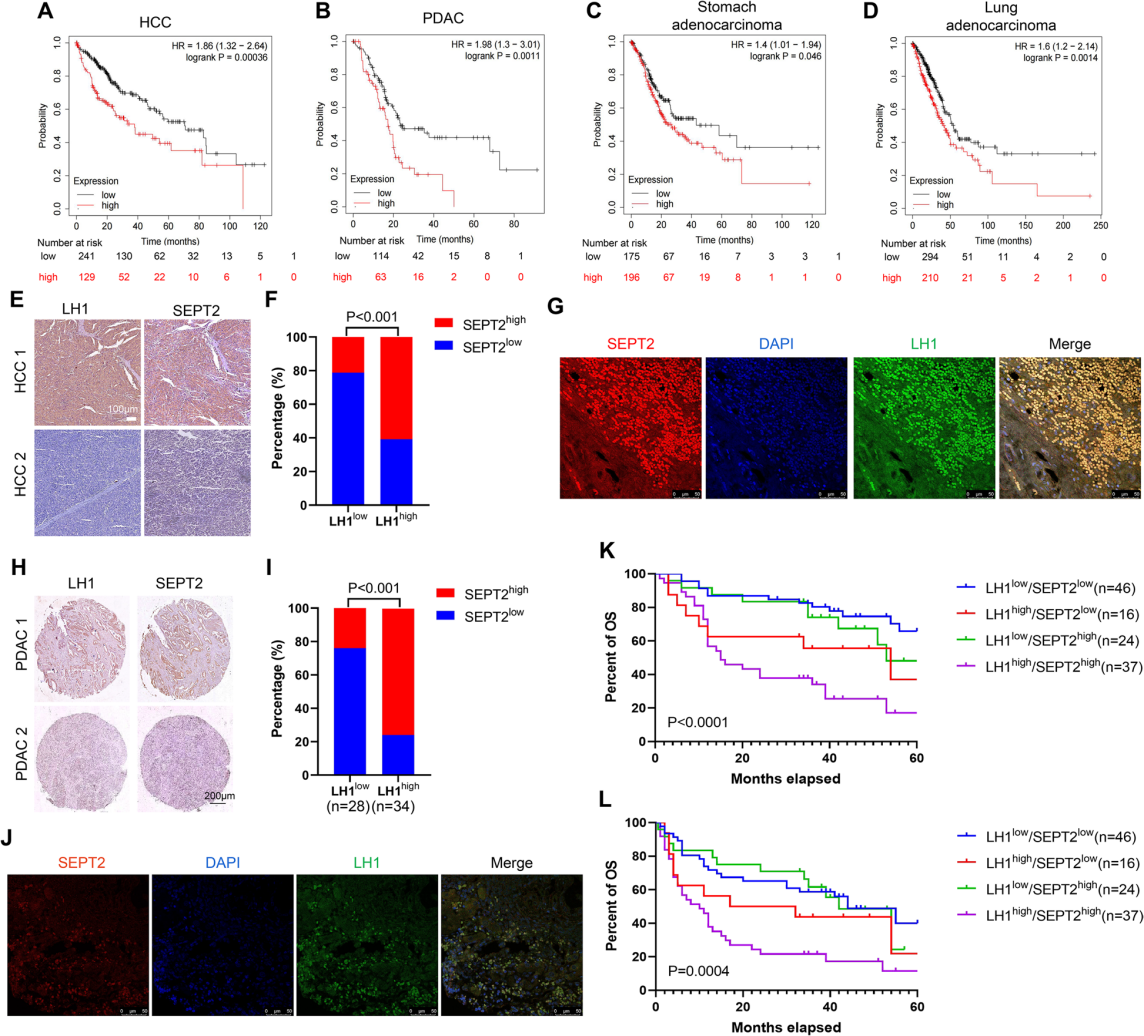
Co-expression of LH1 and SEPT2 correlates with the poorest prognosis of HCC and PDAC patients **A-D** High SEPT2 expression was associated with short OS time in HCC (A), PDAC (B), stomach (C), and lung adenocarcinoma (D) patients using the KMplotter database. **E** Representative IHC images showing the expression of LH1 and SEPT2 in HCC tissues. **F** Correlation between LH1 and SEPT2 expression level in HCC tissues. **G** Double staining of LH1 and SEPT2 in HCC paraffin sections.. **H** Representative IHC images showing the expression of LH1 and SEPT2 in PDAC tissues. **I** Correlation between LH1 and SEPT2 expression level in PDAC tissues. J Double staining of LH1 and SEPT2 in PDAC paraffin sections.. **K**&**L** The LH1^high^/SEPT2.^high^ subgroup HCC patients had the shortest OS (K) and DFS (L) time. *, *P* < 0.05;**, *P* < 0.01;***, *P* < 0.001

Like LH1, we found that high SEPT2 expression level was associated with poor prognosis in HCC (Fig. 7A), PDAC (Fig. 7B), stomach adenocarcinoma (Fig. 7C), and lung adenocarcinoma (Fig. 7D) patients using the KM-Plotter online database. The expression of SEPT2 in HCC and PDAC tissues were then detected by IHC staining. We found a positive correlation between LH1 and SEPT2 expression in HCC tissues (*n* = 153). Chi-square analysis showed that high SEPT2 expression was associated with advanced BCLC tumor stage, microvascular invasion and LH1 expression level (Table 1, Fig. 7E and F). The co-expression of LH1 and SEPT2 was confirmed by IF staining in HCC tissues (Fig. 7G). In addition, high SEPT2 expression levels were slightly correlated with shorter overall survival and disease-free survival with no significant difference (Supplementary Fig. 11A and B). Similarly, we found a positive correlation between LH1 and SEPT2 expression in PDAC tissues (*n* = 62) (Fig. 7H and I). The co-expression of LH1 and SEPT2 was confirmed by IF staining in PDAC tissues (Fig. 7J). PDAC patients with high SEPT2 expression level tended to have shorter OS and DFS (Supplementary Fig. 11C and D). Finally, we grouped the patients into four subgroups according to the expression levels of LH1 and SEPT2. Kaplan–Meier survival analysis showed that the subgroup with both high LH1 and SEPT2 expression had the shortest overall survival time (Fig. 7K) and disease-free survival time (Fig. 7L). In summary, we showed that SEPT2 expression level was positively associated with LH1 in both HCC and PDAC tissues and the combination of these two proteins defined a subgroup with the poorest prognosis.

## Discussion

Most solid tumors are featured by excessive ECM deposition and increased tissue stiffness, especially HCC and PDAC. Severe desmoplasia can be seen in most PDAC tissues. One distinct feature of HCC is that most cases develop on the basis of liver cirrhosis, which means ECM deposition happens before the onset of HCC. Confined migration, defined as the cell migration in confined tracks of pores, is involved in most steps of cancer metastasis, including local infiltration, intravasation, extravasation, and metastatic foci formation at the target organ. Matrix degradation is required for cancer cell migration when the cross-sectional area of interfibrillar pores is less than ~ 7μm^2^ [46]. Biomimetic 3D ECM gels, high-precision microfluidic device, and transwell chambers are the main tools to mimic the in vivo confined migration environments that cancer cells are associated with [8]. In comparison to biomimetic gels and transwell chambers, high-precision microfluidic devices have the advantage of confinement size control and real-time observation. Although biomimetic gels can create a 3D environment to mimic the in vivo situation, it is difficult to monitor real-time cell migration. These tools should be used together to comprehensively study confined migration.

Another important issue is that the molecular mechanism of confined migration of cancer cells remains largely unknown. We report that LH1 is necessary for confined migration of HCC and PDAC cells. LH1-overexpressing cells move faster in confined channels, invaded further in the 3D invasion model, and formed more spheres in stiff hydrogels. We also found that high LH1 expression correlated with advanced tumor stage, and poor prognosis of both HCC and PDAC patients. We should also note that LH1 is a promoter but not a determinant of confined migration, as the PLC/PRF/5 cells with relatively higher LH1 expression did not enter the confined channels. Moreover, neither the proliferation nor unconfined 2D migration were affected by LH1 expression, which was consistent with previous studies [47, 48]. We have also fabricated the single cell confined migration chip containing a single-cell capture structure. Only SNU449 cells and SW1990 cells could enter this channel, which might reflect the relatively small size and the intrinsic biological features of these cells. Our results indicated that LH1 could be a therpeutic candidate in both HCC and PDAC. However, there is currently no specific inhibitor targeting LH1. The LH1 inhibitor used in this study, Dipyridyl, has limited selectivity.

We further elucidated the interaction between LH1 and SEPT2 proteins. LH family members are normally recognized as collagen crosslinkers [49]. However, our immunoprecipitation results did not show the interaction between LH1 and collagen, implying a non-canonical function of LH1. The septin family contains 13 members in total. They can further be divided into four subfamilies: septin2 (septin1/2/4/5), septin3 (septin3/9/12), septin6 and septin7 (septin8/10/11/14). These proteins can self-assemble into hexamers (septin2-6–7-7–6-2) or octamers (septin2-3–6-7–7-6–3-2), and further assembled into filamentous structures [25, 26], which are more stable than actin filaments and microtubules [27]. Increasing studies have shown that the septin family molecules could regulate cytoskeleton, cell polarization, and vesicle transport [25]. Septins are essential for the migration of many cell types, including epithelia [50], fibroblasts, lymphocytes [51] and neurons [52]. For example, both SEPT2 and SEPT9 could enhance maturation of focal adhesions and renal cell migration, but only SEPT9 could directly cross-link pre-polymerized action filaments into bundles [50]. SEPT7 mutation results in defects in actomyosin assembly in Drosophila, and septin proteins alone can bundle actin filaments into rings [53]. In the context of cancer, SEPT9 enables the generation of F-actin bundles which are required for the sustained stabilization of highly contractile actomyosin structures in melanoma [54] and breast cancer [55]. On the other hand, septin cytoskeleton can form a diffusive barrier around nascent podosomes, compartmentalized actin-rich adhesions, and promote their maturation in endothelial cells [56]. We found that LH1 could enhance actin polymerization and knock down of SEPT2 could inhibit the confined migration of LH1-overexpressing cells. Meanwhile, knockdown of SEPT2 could also decrease the F-actin intensity. Interestingly, co-IP experiments showed that SEPT2 could bind to α-actin instead of β-actin protein in HCC cells, suggesting that SEPT2 could directly bundle actin filaments. Although LH1 was predicted to be located in endoreticullum, extracellular space, nucleus, and cytoskeleton based on the immunofluoresence staining results in the Protein Atlas database, we have showed that LH1 was not expressed widely across the cytoplasm.

The first regulator of mammalian septin organization is Borg3, which could bind with SEPT6 and enhance the septin network [57]. We found that LH1 could bind with SEPT2, stabilize its protein abundance, and enhance septin filaments. The hydroxylase domain of LH1 was crucial for the interaction between LH1 and SEPT2, and the loss of this domain diminished its impact on confined migration. These results reflects the hydroxylase domain is necessary for the binding between LH1 and SEPT2, which might be a direct impact on protein binding or the conformational changes after deleting this domain. The exact binding site for LH1-SEPT2 binding should be demonstrated in the future work. Posttranslational modifications such as SUMOylation, acetylation, and phosphorylation, have been reported to affect the septin network [58]. Protein hydroxylation is a posttranslational modification catalyzed by dioxygenases [59]. The hydroxylation modification can take place on various amino acids, including but not limited to proline, lysine, asparagine, aspartate, and histidine. Protein hydroxylation may influence protein stability, protein–protein interaction, kinase activity, or other posttranslational modifications. A classic example is hypoxia inducible factor alpha (HIF-α) prolyl hydroxylation, which leads to HIF-α protein degradation [60]. As a hydroxylase, LH1 could stabilize SEPT2 protein. We found that LH1 could increase the hydroxylation of SEPT2 and prevent the lysosome-meidated degradationo of SEPT2. Another point that should be noted is that there is a lysine residue at the interface between SEPT2 and SEPT6 [61], implying that LH1 might also regulate the interaction between SEPT2 and other septin members. Thus, we proposed that LH1 may enhance the septin network by at least two ways: increasing SEPT2 protein stability and promoting the binding between SEPT2 and SEPT6. Another limitation of this study is that the sample size is relatively small to reveal the prognostic value of LH1 and SEPT2.

## Conclusion

Collectively, we demonstrated that LH1 increased SEPT2 stability, thereby promoting the formation of Septin and F-actin networks, which in turn, promotes the confined migration of HCC and PDAC cells. The current work revealed novel molecular mechanism of LH1 on cancer cell confine migration and metastasis, which is expected to provide new strategy for HCC and PDAC diagnosis or treatment.

## Supplementary Information

**Additional file 1: Supplementary Table 1.** The top 30 un-regulated proteins in HCC tissues.

**Additional file 2: Supplementary Table 2.** The primers used in the study.

**Additional file 3. Supplementary Video 1. **Confined migration of vector and LH1 overexpressed SK-Hep1 cell line.

**Additional file 4. Supplementary Information**.

## Data Availability

All data are available in the main text or the supplementary materials. All data generated in this study can be obtained from the corresponding authors upon reasonable request.

## Abbreviations

HCC: Hepatocellular carcinoma
PDAC: Pancreatic ductal carcinoma
LC–MS: Liquid chromatography-mass spectrometry
Co-IP: Co-immunoprecipitation
LH1: Lysine hydroxylase
SEPT2: Septin2
IHC: Immunohistochemistry
qRT-PCR: Quantitative realtime polymerase chain reaction
POSTN: Periostin
ACSL4: Acyl-CoA Synthetase Long Chain Family Member 4
MCM6: Minichromosome Maintenance Complex Component 6
MCM4: Minichromosome Maintenance Complex Component 4
SMAP: Stromal Membrane-Associated Protein 1
PLIN2: Perilipin 2
G6PD: Glucose-6-Phosphate Dehydrogenase
MCM2: Minichromosome Maintenance Complex Component 2
F-actin: Filamentous actin
FRAP: Fluorescence recovery after photobleaching
