# Supplementary material for: Lysyl hydroxylase LH1 promotes confined migration and metastasis of cancer cells by stabilizing Septin2 to enhance actin network
Source: Mol Cancer. 2023 Jan 31;22:21. doi: 10.1186/s12943-023-01727-9 (PMC9887875; doi:10.1186/s12943-023-01727-9)
Supplement: Supplementary file 4 — Additional file 4. Supplementary Information. [file 12943_2023_1727_MOESM4_ESM.docx]

**Supplementary Information**

**Lysyl hydroxylase LH1 promotes confined migration and metastasis of cancer cells by stabilizing septin2 to enhance actin network**

Zihan Yang^1,2^, Li Zhou^1,3^, Tongxu Si^1,2^, Siyuan Chen^3^, Chengxi Liu^5^, Kelvin Kaki Ng^1,2^, Zesheng Wang^1,2^, Zhiji Chen^3^, Chan Qiu^3^, Guopan Liu^1,2^, Qingliang Wang^4^, Xiaoyu Zhou^1,2^, Liang Zhang^1,2^, Zhongping Yao^5^, Song He^3^, Mengsu Yang^1,2^*, Zhihang Zhou^3,1^*

^1^Department of Biomedical Sciences, and Tung Biomedical Sciences Center, City University of Hong Kong, 83 Tat Chee Avenue, Kowloon, Hong Kong SAR, People's Republic of China.

^2^Department of Precision Diagnostic and Therapeutic Technology, City University of Hong Kong Futian Research Institute, Shenzhen, Guangdong, China

^3^Department of Gastroenterology, the Second Affiliated Hospital of Chongqing Medical University, China.

^4^Department of Pathology, the Second Affiliated Hospital of Chongqing Medical University, China.

^5^State Key Laboratory of Chemical Biology and Drug Discovery, Research Institute for Future Food and Department of Applied Biology and Chemical Technology, The Hong Kong Polytechnic University, Hung Hom, Kowloon, Hong Kong SAR, China

* to whom correspondence should be addressed. E-mail: Zhihang Zhou, zhouzhihang@cqmu.edu.cn; Mengsu Yang, [bhmyang@cityu.edu.hk](mailto:bhmyang@cityu.edu.hk)

**Supplementary Methods**

**Lentivirus infection**

Lentiviruses carrying small hairpin RNA (shRNA) sequence targeting human LH1, SEPT2 (Gene ID: 4735) or the coding sequence of LH1 (Gene ID: 5351) gene, were purchased from Ubigene (GuangZhou, China). Sequences for LH1 shRNA and control were as follows: shRNA-1 (5'-CCCAGAAACACATGCGACTTT-3'), shRNA-2 (5'-GCCGACTATTGACATCCACAT-3'), shRNA-3 (5'-AGAAGAGGGAGCAGATCAATA-3'), and control (5'-AAACGTGACACGTTCGGAGAA-3'). Sequences for SEPT2 shRNA were as follows: shRNA-1 (5'-TGATGGTGGTCGGTGAATCAG -3'), shRNA-2 (5'-CCCAGGACCTTCATTATGAAA-3'), shRNA-3 (5'-GGCGGCACATCATTGATAATA-3'). The lentivirus expressing the fusion gene hACTB-3xGGGGS-mCherry was also from Ubigene (GuangZhou, China). The lentivirus expressing the fusion gene hLH1-3xGGGGS-GFP, the fusion gene hSEPT2-3xGGGGS-mCherry, or luciferase were bought from Syngentech (Beijing, China). The cells were cultured in 6-well plates at 1×10^5^ cells/well, and lentivirus was added into the medium separately. The medium was refreshed after 24 hours. Puromycin (LH1) or G418 (SEPT2) was used to screen the stable cells after 72 hours of infection.

**RNA extraction and real-time quantitative PCR**

Total RNA was extracted by TakaRa MiniBEST universal RNA extraction kit (TakaraBio, Japan). The cDNA was obtained by using PrimeScript RT Master Mix (TakaraBio, Japan) then the qPCR was performed in QuantStudio 12K Flex real-time PCR system (Thermo Fisher, USA) with SYBER Green Master Mix (Applied Biosystems, Thermo Fisher, USA). All PCR primers used are listed in Supplementary Table 2.

**Scanning Electron Microscopy (SEM)**

The samples were washed three times by Milli-Q water. The specimens were quickly frozen using liquid nitrogen and then placed in a freezing dryer for 72 hours. The dried samples were stored in the refrigerator for subsequent tests. The surface morphology of the different GelMA were characterized by QUATTRO S SEM in low vacuum mode (Thermo, USA). Low vacuum can help observe the surface topography without sputter coating. The intact GelMA was crushed into small pieces and placed inside the SEM chamber. A partial vacuum was created within the chamber. The images were taken from a distance around 10.0 mm with an acceleration voltage of 10.00 kV.

**Non-scratching wound healing assay**

Non-scratching wound healing assay was performed in the Ibidi culture insert (Ibidi^®^, Munich, Germany), which consisted of two cell culture chambers separated by an interval with the width of 500μm. 70 µL of cell suspension at a density of 5 × 10^5^ cells/ml with complete medium were seeded into each chamber. After 8 hours, the cells completely adhered to the chamber bottom and we removed the insert. We then added fresh complete medium to the well and set it under a real-time tracking microscope (LS720, Etaluma, USA) for 24 hours to observe wound closure.

**Transwell cell migration assay**

2.5×10^4^ cells were plated into each upper chamber with 8-μm pores of 24-well transwell chambers (BD Falcon, Franklin Lakes, NJ, USA) and cultured in 200μL serum-free medium. The bottom chambers were filled with 500μL complete medium. After 24 hours of incubation at 37°C, cells migrated to bottom chamber were fixed with 10% Neutral Buffered Formalin (NBF) and stained with 0.1% crystal violent. The whole membrane images were captured by microscope (Nikon, Ti2-E, Japan) and 5 distinct areas were counted.

**Cell proliferation assay**

Cells were seeded into adherent 96-well plates with 2000 cells per well and three replications for each sample. Cell proliferation was measured every 24 hours for the following five days by using Cell Counting Kit-8 (CCK-8) (MedChemExpress, USA). 450nm was used on the microplate reader (BioTek, Agilent, USA) to measure the absorbance of each well.

**Colony formation assay**

Five hundred cells were seeded into each well of 6-well plates. After 15 days of culture, colonies were fixed by 10% NBF and stained with 0.1% crystal violate (Invitrogen, Carlsbad, CA, USA).

**Western blotting**

Whole-cell lysates were extracted by RIPA lysis buffer and was added to 1:4 ratio loading buffer. Then, samples were separated by 10% SDS-PAGE, transferred to polyvinylidene fluoride membranes (Millipore, Billerica, MA, USA) and followed by using semi-dry transfer apparatus (Bio-Rad, German). Blots were blocked in fast blocking reagent (Beyotime, Shanghai, China) for 15mins at room temperature and then incubated with antibodies against LH1(Thermo Fisher, USA), SEPT2 (Abclonal, China), SEPT3 (Abclonal, China), SEPT6 (Abclonal, China), SEPT7 (Abclonal, China), α-actin (Abclonal, China), β-actin (Abclonal, China), and GAPDH (Abclonal, China) at 4 °C overnight. The HRP-coupled anti-rabbit or mouse secondary antibody (Beyotime, Shanghai, China) were used with 1:1000 ratio dilution rate for 2 hours of incubation. To visualize results, we applied chemiluminescence reagent (ECL, Beyotime, Shanghai, China) to membranes and provided exposure to ChemiDoc (Bio-Rad, Germany). For SEPT2 degradation assay, cells were plated in 6-well plates and treated with CHX (40μg/ml; Sigma-Aldrich, USA) for different time durations (0,6,12,24 hours). To determine the degradation pathway, the cells were simutaneously treated with chloroquine (50μM; Sigma-Aldrich, USA) or MG132 (10μM; Sigma-Aldrich, USA).

**Immunohistochemistry (IHC)**

This was done according to previous work [28]. Following deparaffinization and rehydration, tissue samples (4μm slices) were incubated in 0.3% H_2_O_2_ in methanol for 30 min at 37 °C to block endogenous peroxidase. The sections were then boiled in 10 mmol/L citrate buffer (pH 6.0) for 2 min in an autoclave. The sections were incubated with anti-LH1 (Abcam, UK) or anti-SEPT2 (Abclonal, China) antibodies overnight at 4 °C, incubated with HRP-conjugated anti-rabbit secondary antibody (ZSGB-BIO, China), and visualized with DAB. The negative control was only incubated with secondary antibody. The intensity of staining (brown color) was semi-quantitatively scored as follows: 1-weak; 2-medium; 3-strong; and 4-very strong. The percentage of maximally stained tumor cells in each section was recorded (0-<5%; 1-5–30%; 2-30–50%; 3->50%). High expression of target proteins was defined as a combined score for the intensity and area of staining that was larger than the mean scores (LH1 score ≥6; SEPT2≥4). The results were verified by two independent pathologists.

**HE staining**

The 4 μm-thick sections were first deparaffinated and then rehydrated. They were stained with hematoxylin and eosin successively. Finally, the sections were mounted with Permount TM mounting medium and subjected to pathological analysis.

**Immunofluorescence (IF) assay**

Cells were fixed with 10% Neutral Buffered Formalin (NBF) for 15 min and 0.25% triton (Sigma-Aldrich, USA) in Phosphate buffer saline (PBS) for 10 mins at 4 °C for permeabilization. After washing with PBS three times, we blocked the sample using 1% bovine serum albumin (Sigma-Aldrich, USA) in PBS for 1 hour. Cells were then incubated with the primary antibody against LH1 (Abcam, USA) or SEPT2 (Abclonal, China) overnight at 4 ℃. After 3 more washes with PBS, cells were then incubated with fluorescence-labeled secondary antibody for 1 hour at room temperature. The nuclei were stained by Hochest 33342 (Thermo Fisher, USA) for 5 mins. The F-actin was stained with 647-labeled phalloidin (Abcam, USA). The endoreticullum were stained with ER-tracker Red (Beyotime, China). For paraffin immunofluorescence double staining, the paraffin-embeded sections were deparaffinized, rehydrated and blocked with control serum. The sections were then incubated with LH1 antibody and then corresponding secondary antibody. After the second-round block, the sections were then incubated with SEPT2 antibody and then corresponding secondary antibody. Images were captured by high-speed confocal microscope (Nikon, A1HD25, Japan).

**Supplementary Figures**


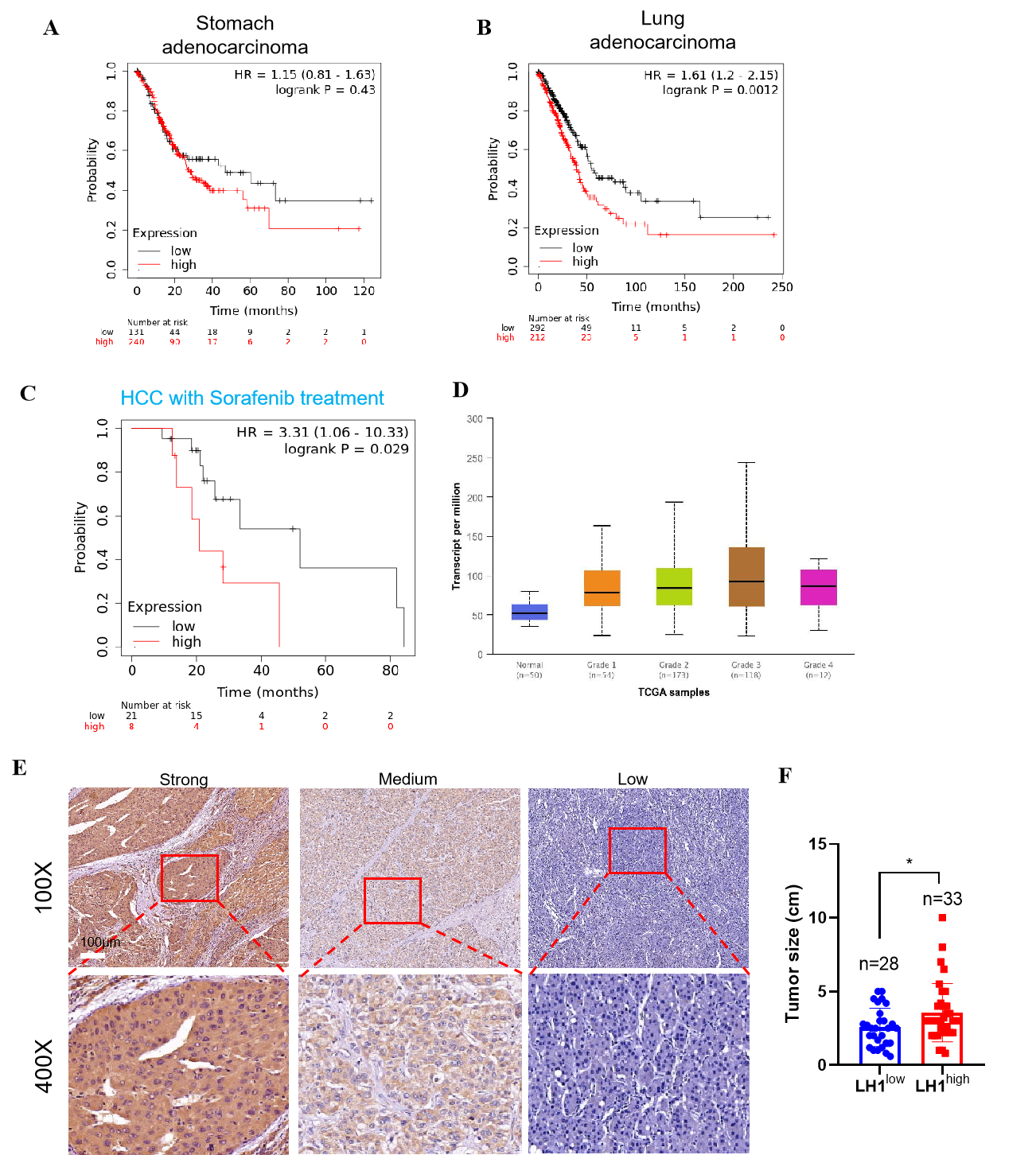


**Supplementary Fig. 1.** The expression of LH1 in HCC and PDAC tissues **A&B** High SEPT2 expression was associated with short OS time of patients with stomach (A) or lung adenocarcinoma (B) in the KMplotter database. **C** Kaplan-Meier survival analysis showing the prognostic value of LH1 in HCC patients with Sorafenib treatment from KMplotter. **D** UALCAN data showing the expression level of LH1 in HCC tissues with different grade. **E** IHC images showing the different expression of LH1 in HCC tissues. **F** The tumor size was larger in LH1^high^ group than LH1^low^ group in PDAC patients. *, P<0.05;**, P<0.01;***, P<0.001


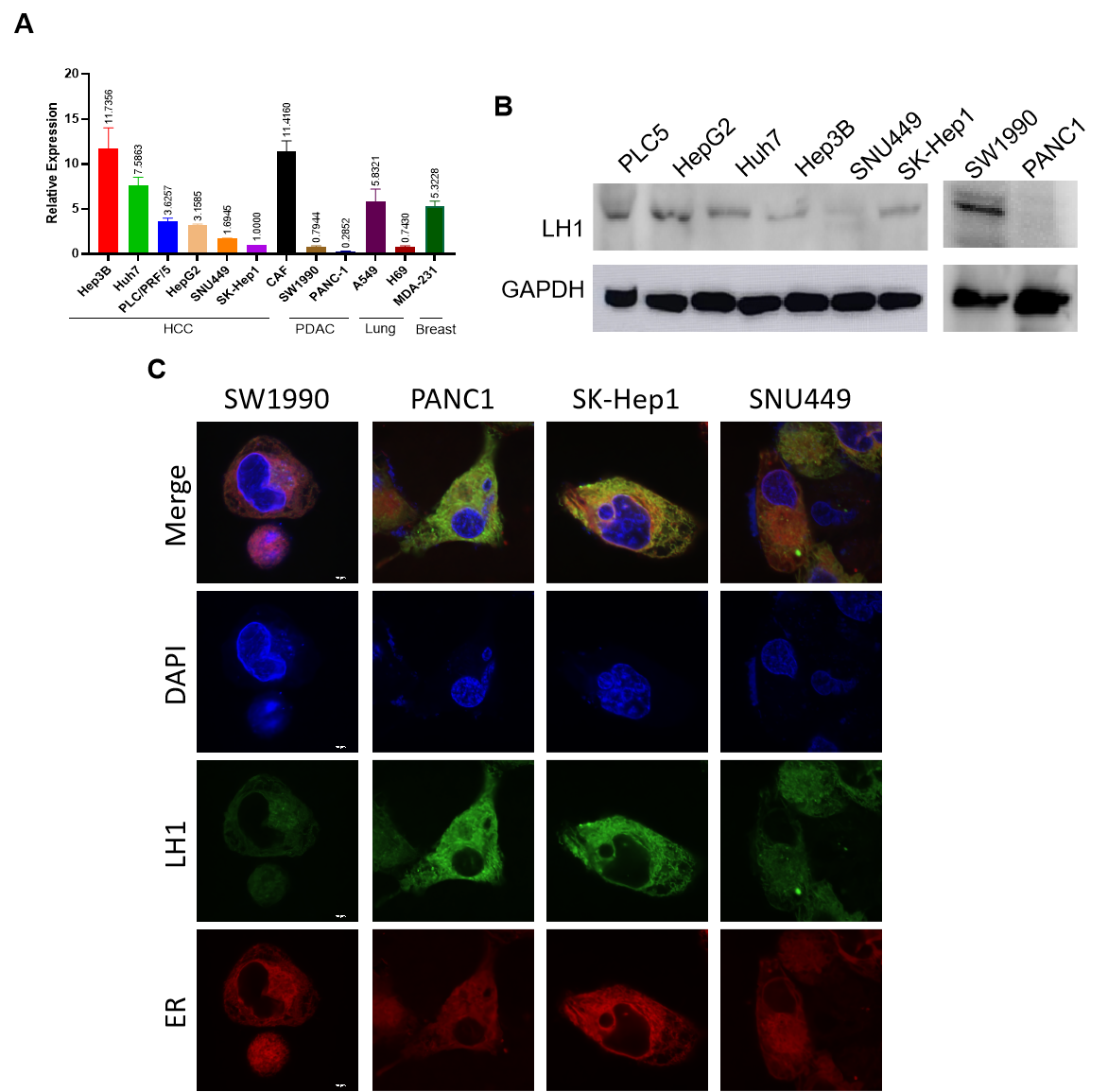


**Supplementary Fig. 2. The exprssion of LH1 and SEPT2 in HCC and PDAC cells. A** Differential expression level of LH1 in HCC, PDAC, lung adenocarcinoma and breast cancer cell lines by RT-PCR. **B** Expression level of LH1 in HCC and PDAC cell lines by Western blot. C. ER location of SW1990, PANC1, SK-Hep1 and SNU449.


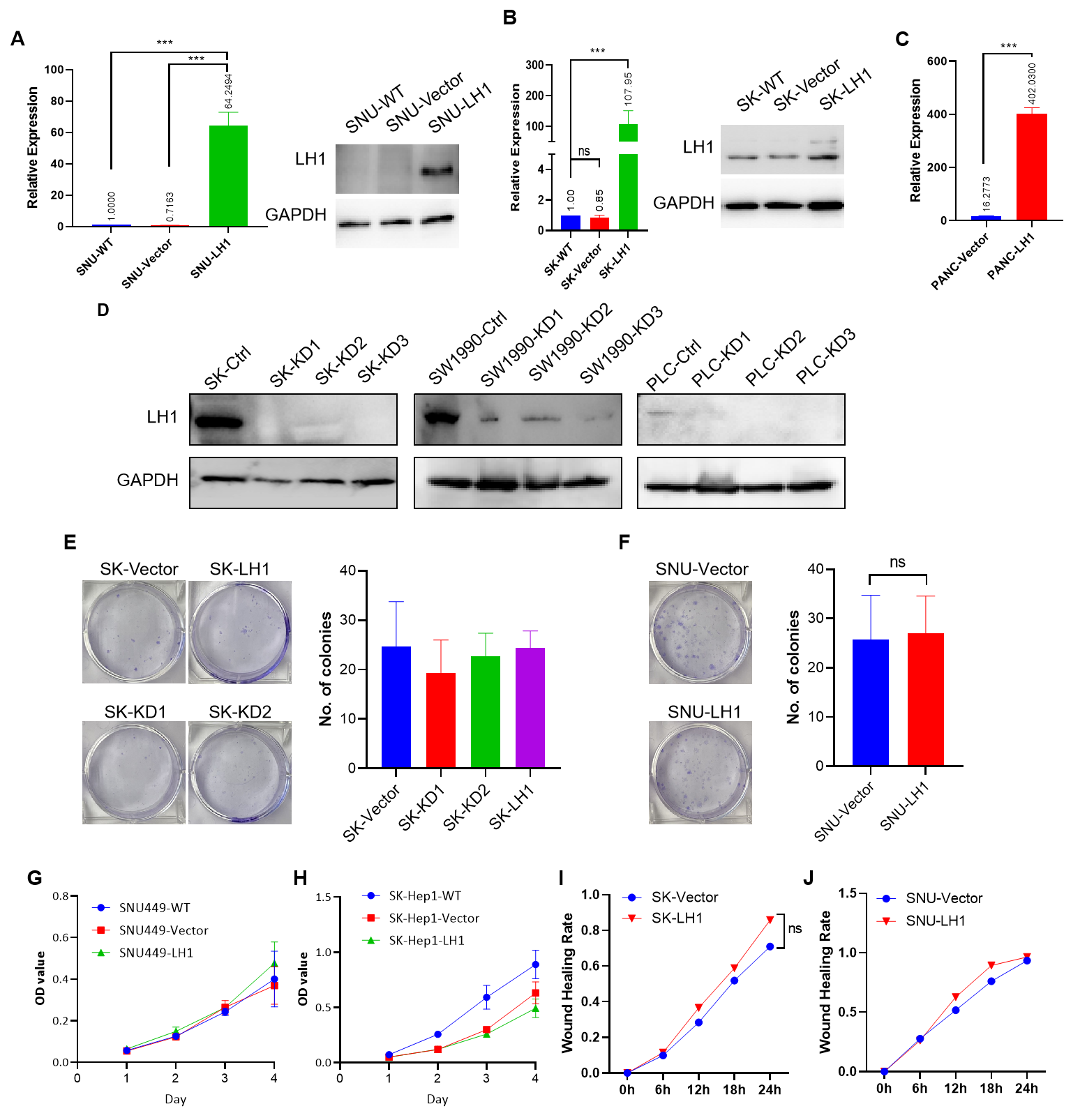


**Supplementary Fig. 3.** LH1 does not affect cell proliferation and unconfined migration **. A-C** Validation of LH1 overexpression in SNU449 (C), SK-Hep1 (D) and PANC1 (E) cells by RT-PCR and Western blot. **D** Validation of LH1 knockdown in SK-Hep1, SW1990, and PLC/PRF/5 cells by Western blot. **E** Colony formation of SK-Hep1 cells after knockdown or overexpression of LH1. **F** Colony formation of SNU449 cells after overexpression of LH1. **G** Proliferation curve of wild type, control and LH1-overexpressing SNU449 cells. **H** Proliferation curve of wild type, control and LH1-overexpressing SK-Hep1 cells. **I** Wound healing rate of control and LH1-overexpressing SK-Hep1 cells. **J** Wound healing rate of control and LH1-overexpressing SNU449 cells. *, P<0.05;**, P<0.01;***, P<0.001


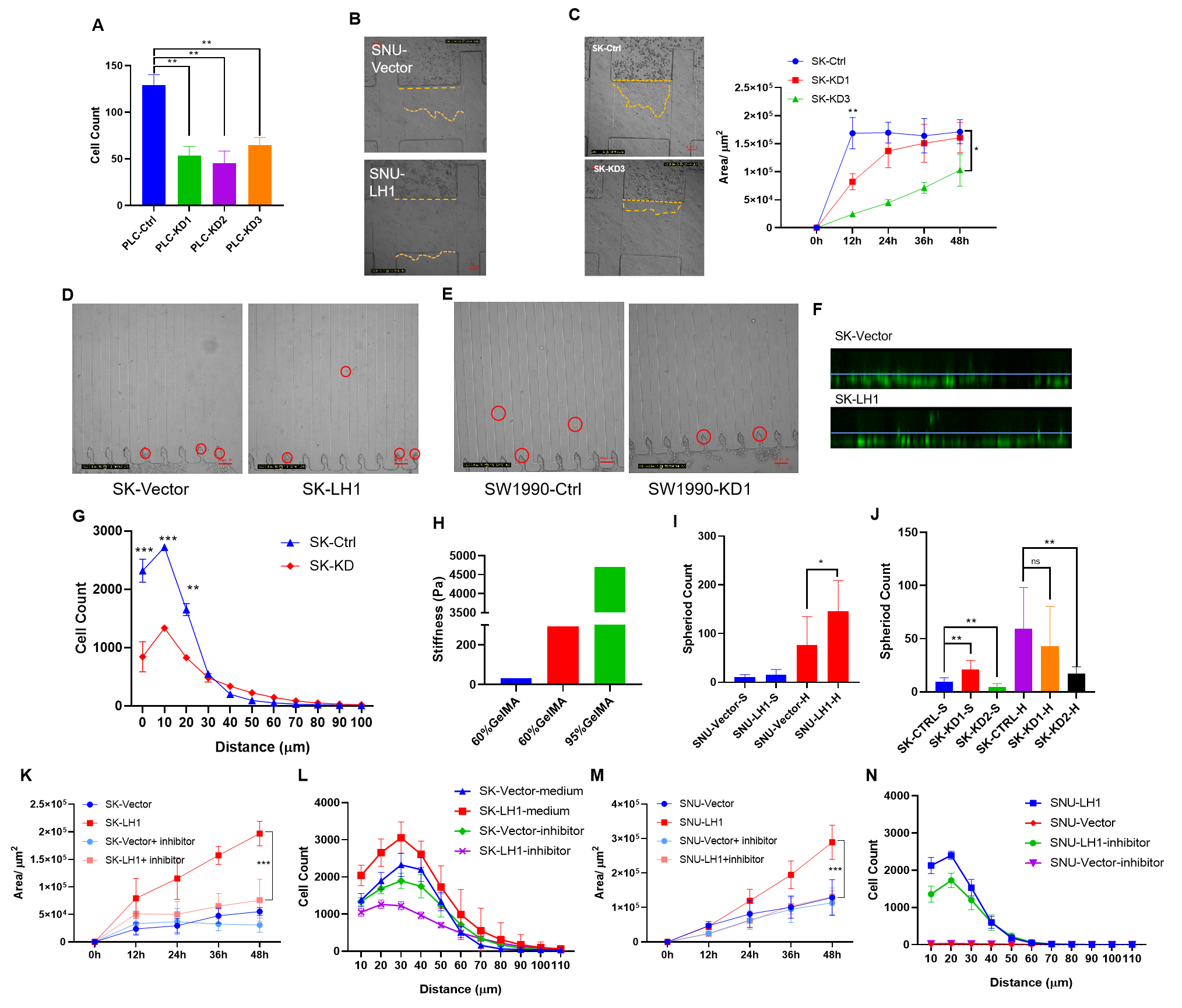


**Supplementary Fig. 4.** Knockdown of LH1 inhibits cancer cell confined migration **A** Transwell migration of PLC/PRF/5 cells after LH1 knockdown. **B** Representative images showing the collective confined migration of SNU449 cells after LH1 overexpression. **C** 2D confined migration of SK-Hep1 after LH1 knockdown. **D&E** Images of single cell confined migration of SK-Hep1 after LH1 overexpression (D) or SW1990 after LH1 knockdown (E). **F** Representative images showing the 3D vertical invasion of LH1-overexpressing SK-Hep1 cells. **G** 3D invasion was inhibited by LH1 knockdown in SK-Hep1 cells. **H** Stiffness of the GelMA gels with different substitution and UV exposure time. **I** Sphere numbers of LH1-overexpressing and control SNU449 cells under soft (S) or hard (H) gels. **J** 3D sphere formation of SK-Hep1cells with or without LH1 knockdown. **K&L** Inhibitory effect of LH1 inhibitor on 2D confined migration (K) and 3D invasion (L) of LH1-overexpressing SK-Hep1. **M&N** Inhibitory effect of LH1 inhibitor on 2D confined migration (M) and 3D invasion (N) of LH1-overexpressing SNU449 cells. *, P<0.05;**, P<0.01;***, P<0.001


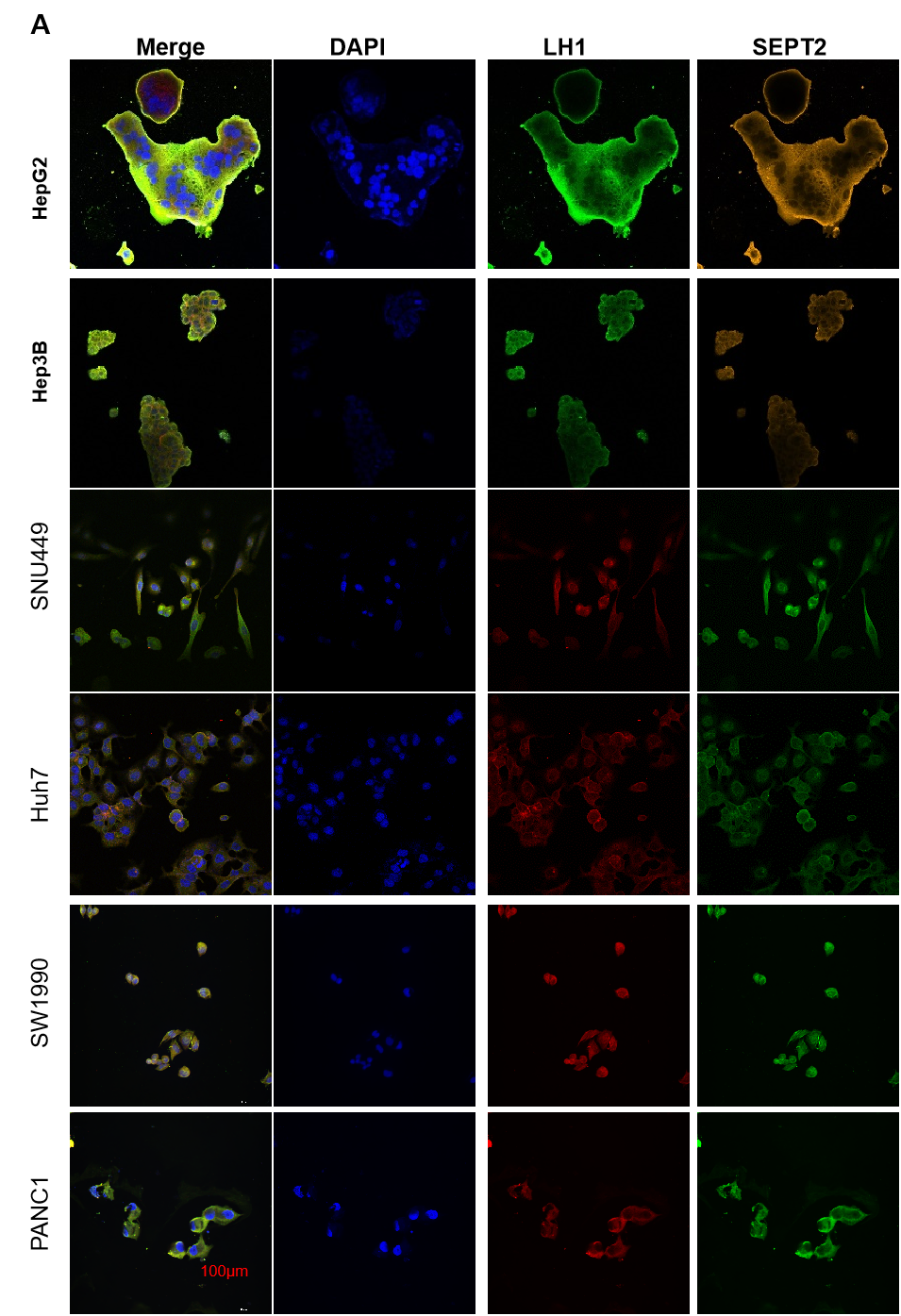


**Supplementary Fig. 5.** Co-localization of LH1 and SEPT2 in HCC and PDAC cells. **A** Co-localization of LH1 and SEPT2 in HepG2, Hep3B, SNU449, Huh7, SW1990 and PANC1 cells.


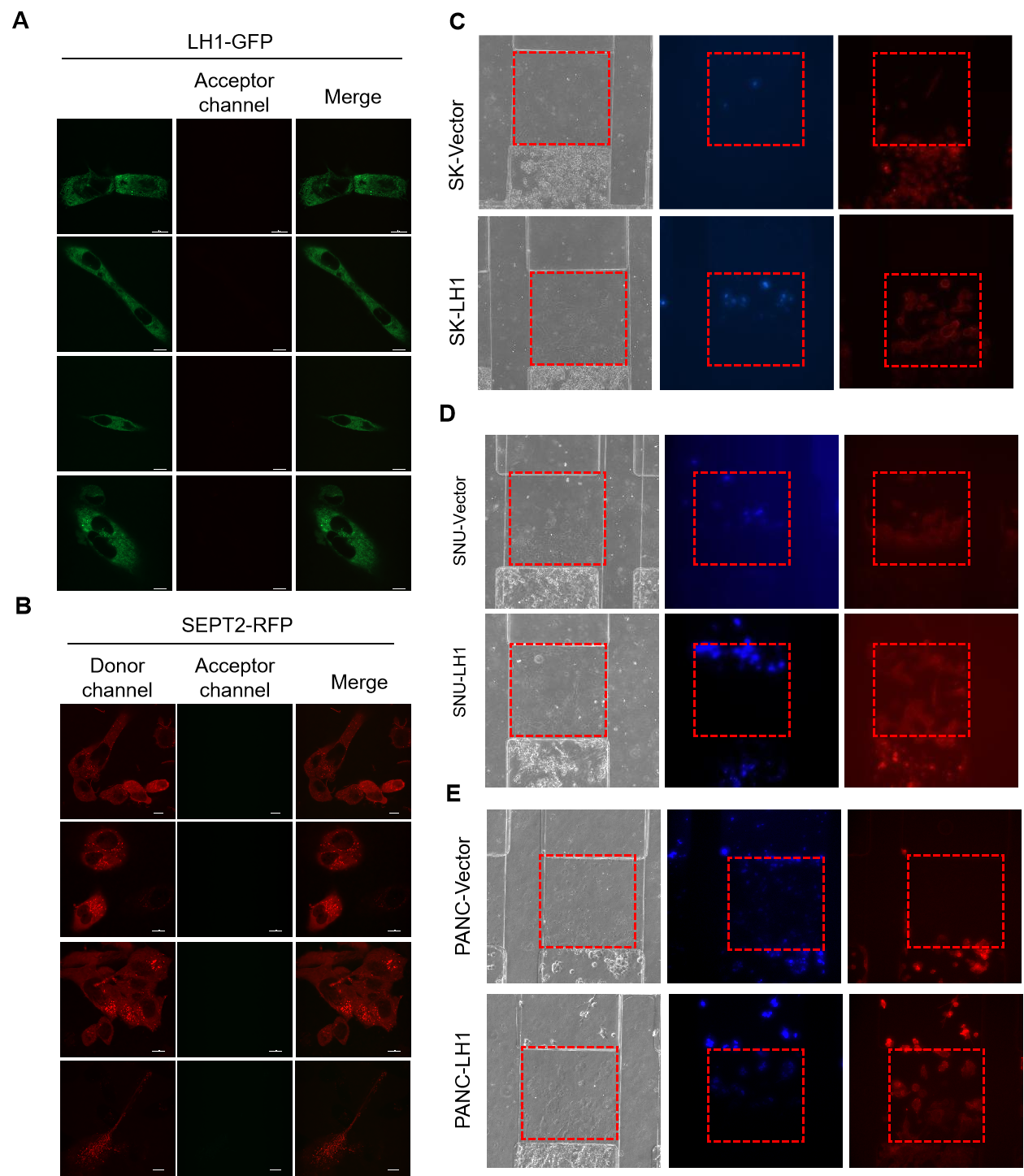


**Supplementary Fig. 6. LH1 facilitated actin polymerization under confined condition. A&B Negative control of FRET assays. B-D** F-actin staining in SK-Hep1 (B), SNU449 (C) and PANC1 (D) cells with or without LH1 overexpression on confined migration channels.


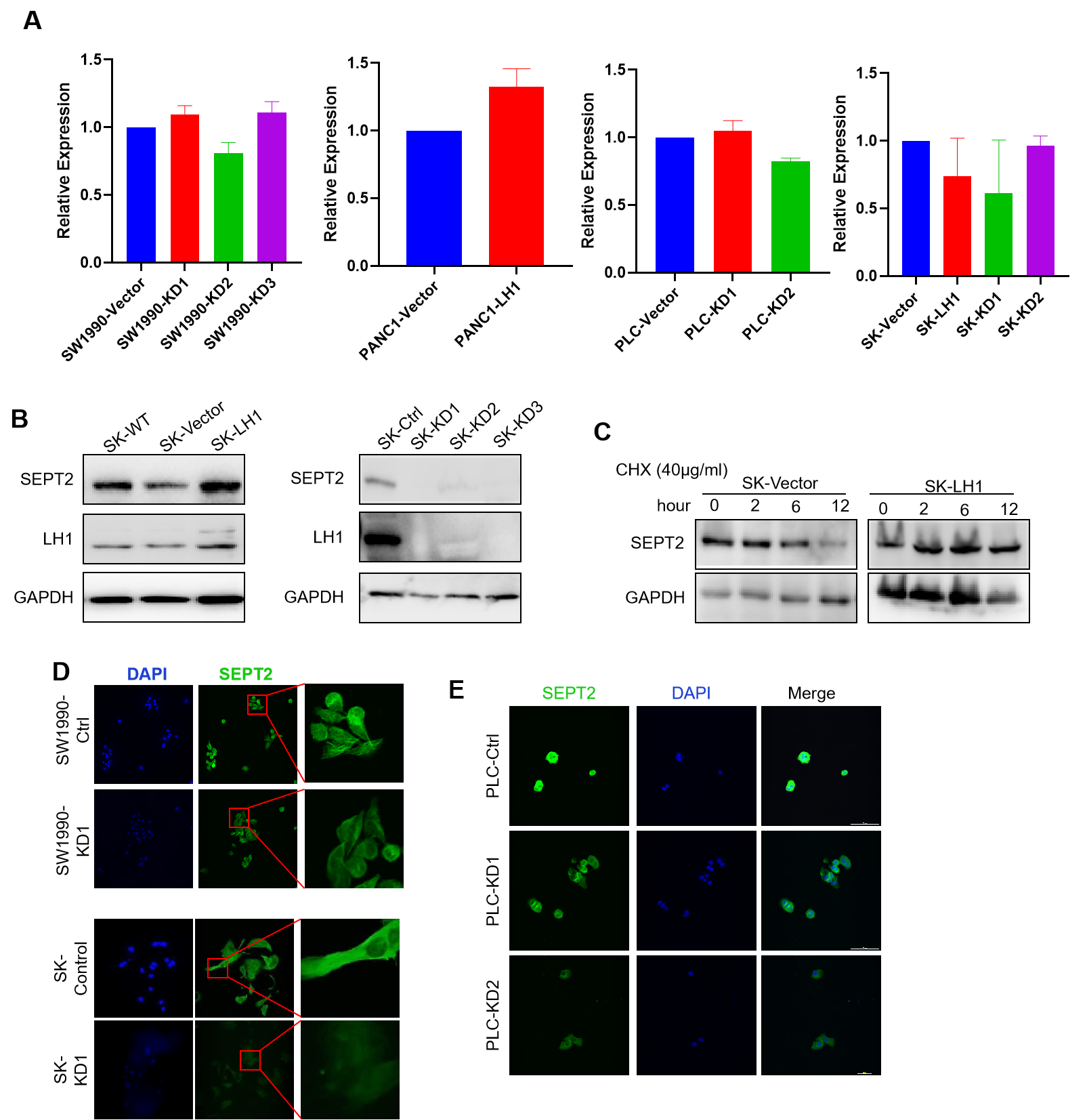


**Supplementary Fig. 7.** LH1 reduces the SEPT2 protein degradation and enhances septin network. **A** RT-PCR results showing that the mRNA level of SEPT2 was not significantly affected by overexpression or knockdown of LH1 in HCC and PDAC cells. **B** Western blot showing the expression of SEPT2 in LH1-overexpression or LH1-knockdown SK-Hep1 cells. **C** The degradation of SEPT2 in LH1-overexpression SNU449 cells. D. The Septin networks in LH1-knockdown HCC and PDAC cells. E. F-actin staining showing reduction in F-actin after LH1 knockdown in PLC/PRF/5 cells.


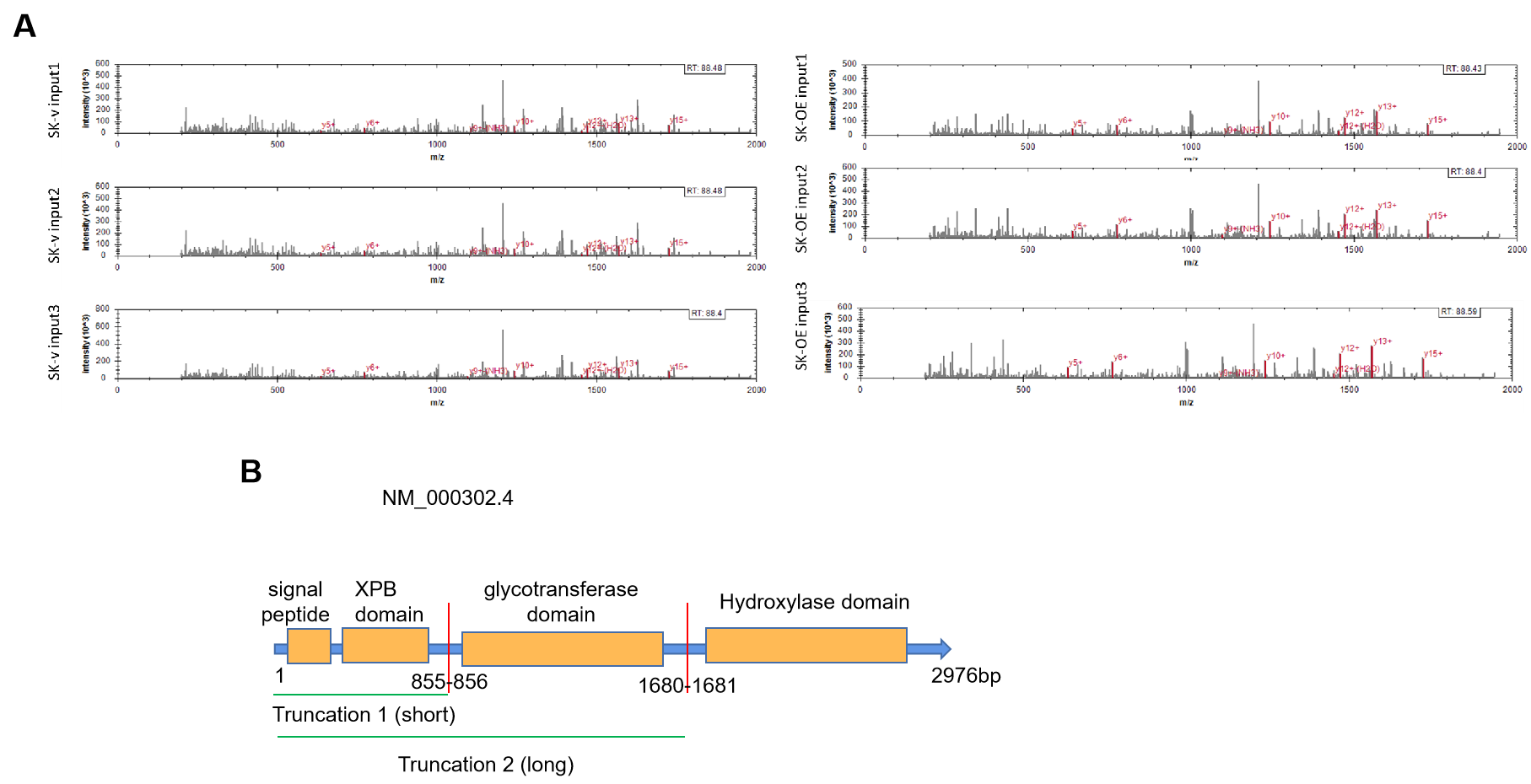


**Supplementary Fig. 8.** LH1 increased SEPT2 hydroxylation. **A** Intensity of SEPT2 hydroxylation in control or LH1-overexpression cells. **B** The domains of LH1 protein and the long truncation (L) and the short truncation (S).

**
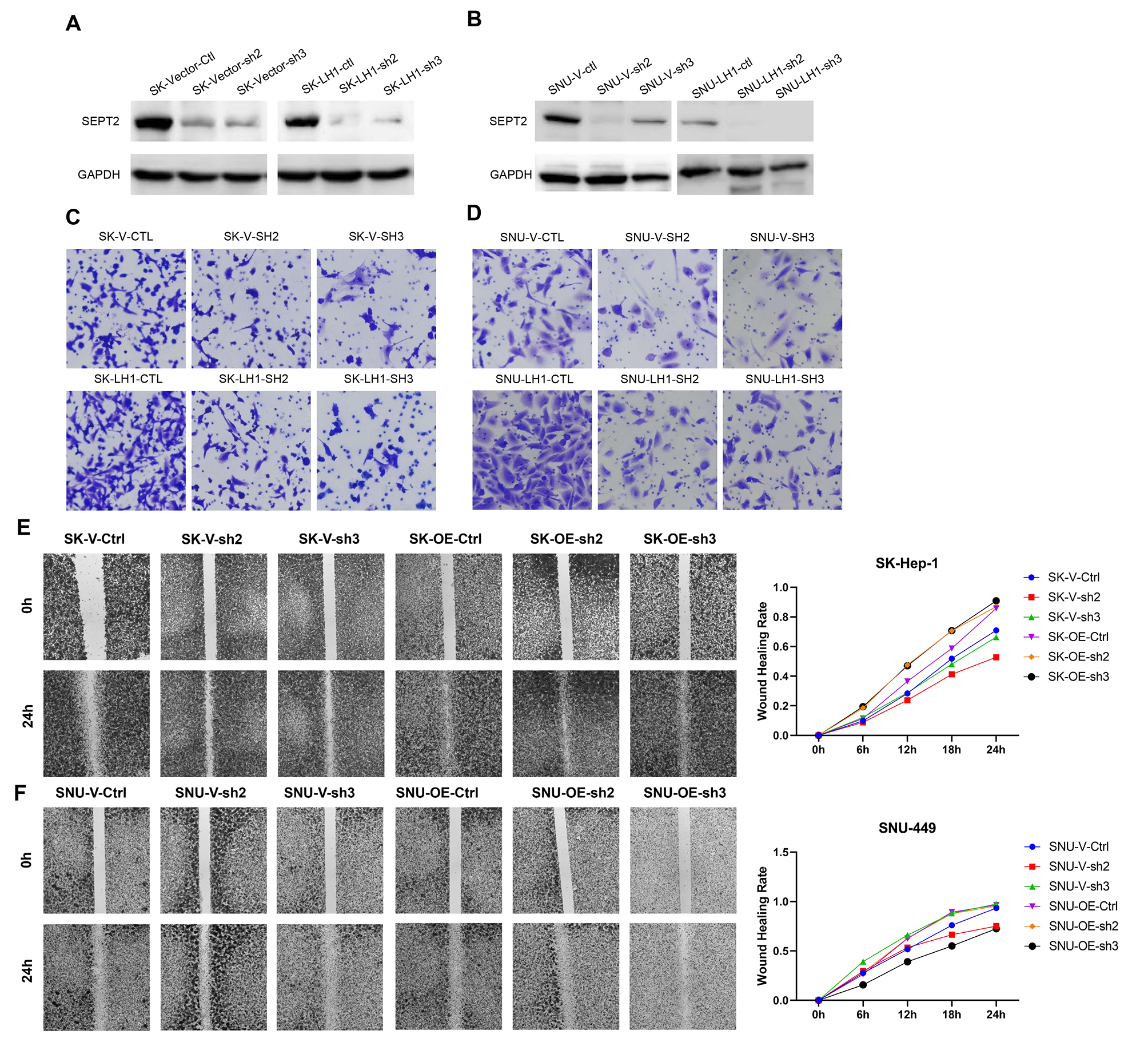
**

**Supplementary Fig. 9.** Knockdown of SEPT2 inhibits confined migration **A** Western blot confirmed the knockdown of SEPT2 in control or LH1-overexpression SK-Hep1 cells. **B** Western blot confirmed the knockdown of SEPT2 in control or LH1-overexpression SNU449 cells. **C&D** Transwell migration image of the indicated cells. **E&F** Wound healing of the indicated cells, especially the knockdown of SEPT2 (sh2/sh3) in LH1-overexpression (oe) cells.


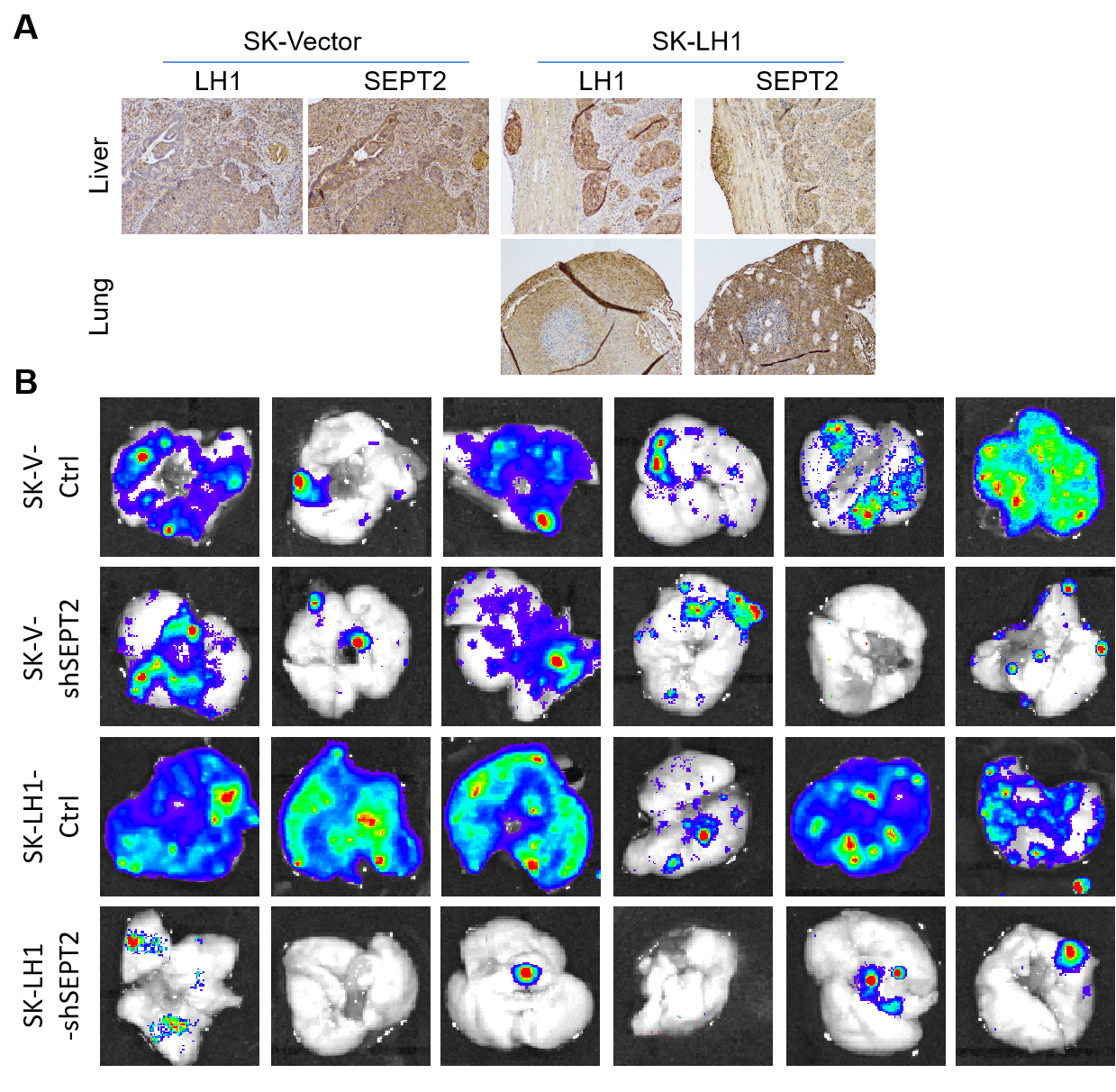


**Supplementary Fig. 10. SEPT2 mediates the pro-metastasis function of LH1. A** Representative IHC images showing the expression of LH1 and SEPT2 in PDAC tissues. **B** Knock-down of SEPT2 dramatically reduced the lung metastasis in LH1-overexpression cells, with slight effect in control cells.


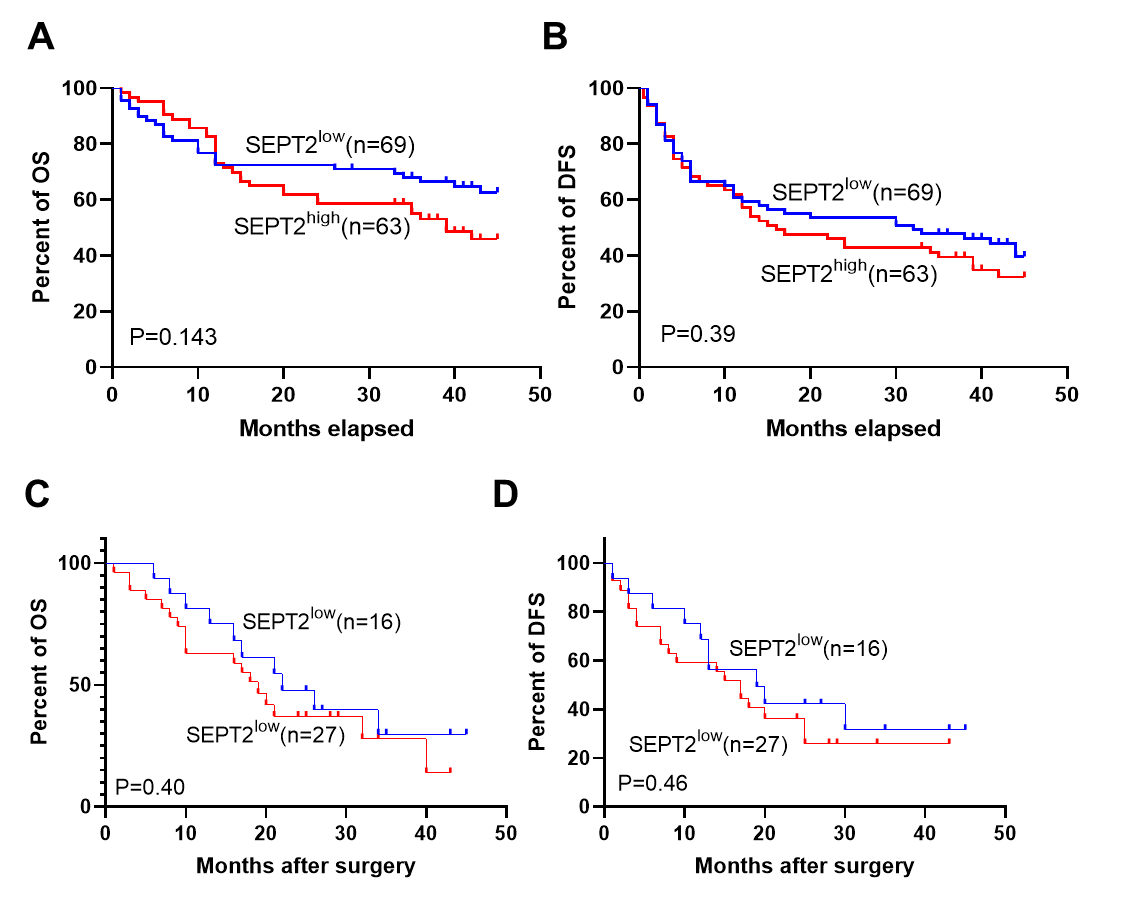


**Supplementary Fig 11. The prognostic value of SEPT2 in HCC and PDAC. A** OS of SEPT2^high^ and SEPT2^low^ HCC patients. **B** DFS of SEPT2^high^ and SEPT2^low^ HCC patients. **C** OS of SEPT2^high^ and SEPT2^low^ HCC patients after surgery. **D** DFS of SEPT2^high^ and SEPT2^low^ HCC patients after surgery.
